# Open resource of clinical data from patients with pneumonia for the prediction of COVID-19 outcomes via deep learning

**DOI:** 10.1038/s41551-020-00633-5

**Published:** 2020-11-18

**Authors:** Wanshan Ning, Shijun Lei, Jingjing Yang, Yukun Cao, Peiran Jiang, Qianqian Yang, Jiao Zhang, Xiaobei Wang, Fenghua Chen, Zhi Geng, Liang Xiong, Hongmei Zhou, Yaping Guo, Yulan Zeng, Heshui Shi, Lin Wang, Yu Xue, Zheng Wang

**Affiliations:** 1 Key Laboratory of Molecular Biophysics of Ministry of Education, Hubei Bioinformatics and Molecular Imaging Key Laboratory, Center for Artificial Intelligence Biology, College of Life Science and Technology, Huazhong University of Science and Technology, Wuhan, China; 2 Department of Clinical Laboratory, Union Hospital, Tongji Medical College, Huazhong University of Science and Technology, Wuhan, China; 3 Research Center for Tissue Engineering and Regenerative Medicine, Union Hospital, Tongji Medical College, Huazhong University of Science and Technology, Wuhan, China; 4 Department of Respiratory and Critical Care Medicine, Liyuan Hospital, Tongji Medical College, Huazhong University of Science and Technology, Wuhan, China; 5 Department of Critical Care Medicine, Renmin Hospital of Wuhan University, Wuhan, China; 6 Department of Radiology, Union Hospital, Tongji Medical College, Huazhong University of Science and Technology, Wuhan, China; 7 Hubei Province Key Laboratory of Molecular Imaging, Wuhan, China; 8 Department of Laboratory Medicine, Liyuan Hospital, Tongji Medical College, Huazhong University of Science and Technology, Wuhan, China; 9 Department of Radiology, Liyuan Hospital, Tongji Medical College, Huazhong University of Science and Technology, Wuhan, China; 10 Department of Gastrointestinal Surgery, Union Hospital, Tongji Medical College, Huazhong University of Science and Technology, Wuhan, China

**Keywords:** Microbiology, Diseases, Health care, Biological techniques, Computational biology and bioinformatics

## Abstract

Data from patients with coronavirus disease 2019 (COVID-19) are essential for guiding clinical decision making, for furthering the understanding of this viral disease, and for diagnostic modelling. Here, we describe an open resource containing data from 1,521 patients with pneumonia (including COVID-19 pneumonia) consisting of chest computed tomography (CT) images, 130 clinical features (from a range of biochemical and cellular analyses of blood and urine samples) and laboratory-confirmed severe acute respiratory syndrome coronavirus 2 (SARS-CoV-2) clinical status. We show the utility of the database for prediction of COVID-19 morbidity and mortality outcomes using a deep learning algorithm trained with data from 1,170 patients and 19,685 manually labelled CT slices. In an independent validation cohort of 351 patients, the algorithm discriminated between negative, mild and severe cases with areas under the receiver operating characteristic curve of 0.944, 0.860 and 0.884, respectively. The open database may have further uses in the diagnosis and management of patients with COVID-19.

## Abstract (web summary)

An open resource comprising chest computed tomography images and 130 clinical features of 1,521 patients with pneumonia, including COVID-19 pneumonia, facilitates the prediction of morbidity and mortality outcomes via deep learning.

## Main

In December 2019, an outbreak of an unknown viral pneumonia severely affected Wuhan, China. The virus was quickly identified and named severe acute respiratory syndrome coronavirus 2 (SARS-CoV-2)^1–3^ by the World Health Organization, and the resulting viral pneumonia is referred to as coronavirus disease 2019 (COVID-19) pneumonia^4–8^. By the end of March, 2020, nearly 200 countries and regions had been affected, with more than 500,000 confirmed cases, and the number of cases is still increasing. This severe situation underscores the urgency for developing effective measures to control the pandemic.

Early diagnosis of patients with COVID-19 pneumonia for timely treatment is critical for containing outbreaks, especially in epidemic regions^2,9–12^. However, this remains a challenging task. Limited medical resources and the large number of patients across many regions affected by COVID-19 commonly result in long waiting times for diagnosis and medical decisions such as quarantine or hospitalization, which potentially increase the chances of cross-infection and lead to poor prognosis. Although confirmation of a COVID-19 diagnosis relies on detection of SARS-CoV-2 RNA by quantitative PCR with reverse transcription (RT–PCR), this test has been found to show a high specificity^9^ (Sp) but a low sensitivity (Sn), with a reported positive rate^13^ of 38–57%.

In addition to aetiological laboratory confirmation, other key diagnostic elements that facilitate identification of COVID-19 pneumonia include clinical features (CFs) and chest computed tomography (CT) imaging^14,15^. Consistent with the importance of these diagnostic elements, the Guidance for COVID-19 (6th edition)^16^ released by the National Health Commission of China uses some of these diagnostic elements to define mild, regular, severe and critically ill forms of confirmed and suspected cases of COVID-19 pneumonia^16–19^. Despite far from complete understanding, studies have begun to reveal relevant CFs, including symptoms of COVID-19 such as fever, dry cough, myalgia and shortness of breath^7,10,11^. Other CFs, such as lymphopenia, elevated levels of inflammatory cytokines and reduction in T cell subsets, are also frequently found^11,12^. Moreover, chest CT imaging characteristics of infected lungs reportedly include ground-glass opacity (GGO) and severity-correlated consolidation^20^. Although the picture remains incomplete, comprehensively pooling the features of the aforementioned diagnostic elements might collectively improve the accuracy and efficacy of diagnosis.

During the ongoing COVID-19 pandemic, the availability of first-hand CT and clinical datasets will be essential and important to help guide clinical decision making, to supply information to deepen understanding of this viral infection, and to provide a basis for systemic modelling that may facilitate early diagnosis for timely medical intervention. One way to achieve this goal is to create an open-access and comprehensive resource containing chest CT images and CFs of individual patients, to facilitate international joint efforts to combat COVID-19 pneumonia.

From the accumulated data in our hospitals, we prepared two cohorts that include a total of 1,170 and 351 individuals, respectively, comprising patients with laboratory-confirmed COVID-19, COVID-19-negative (control) individuals and individuals with suspected COVID-19, and collected their corresponding chest CT images, CFs and SARS-CoV-2 laboratory test results if available. Then, we developed a patient-centric resource, which we named integrative CT images and CFs for COVID-19 (iCTCF) to archive and share the rich data. Using cohort 1, we integrated the highly heterogeneous CT and CF datasets, and built an engineering framework of hybrid learning for unbiased prediction of COVID-19 patients (HUST-19) to predict clinical outcomes, including morbidity outcomes (defined as mild or regular form (type I) and severe or critically ill form (type II)) and mortality outcomes. For morbidity outcomes, the area under the curve (AUC) values of HUST-19 are 0.978, 0.921 and 0.931 for predicting negative cases (control), mild or regular (type I) and severe or critically ill (type II) patients, respectively. We used cohort 2 as a validation dataset to evaluate HUST-19, with consistently promising accuracy. For mortality outcomes, we merged the two cohorts and achieved an AUC value of 0.856 for predicting deceased cases. Using HUST-19, we conducted a retrospective analysis of 299 suspected cases in cohort 1, and predicted 207 potential type I cases and 71 potential type II cases. In conclusion, this resource can be useful for retrospective analysis purposes and for improving the diagnosis and treatment of patients with COVID-19. We have made HUST-19 and iCTCF freely available for academic research at http://ictcf.biocuckoo.cn.

## Results

### A summary of collected datasets

**Fig. 1:**
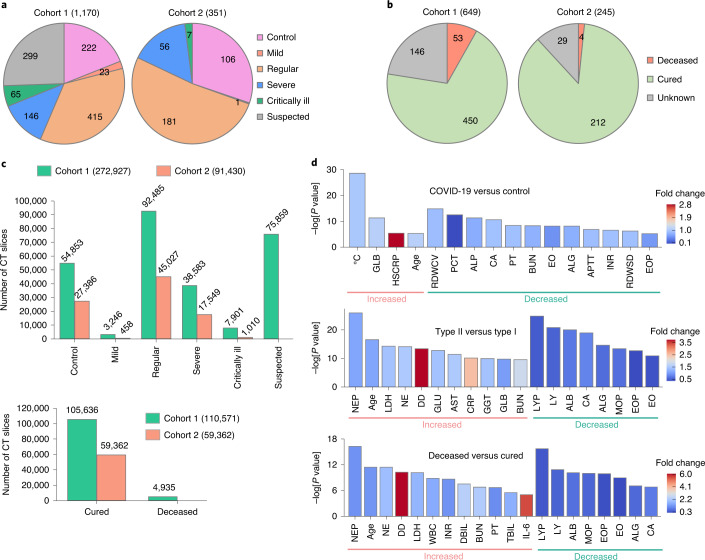
Statistics of the study data. **a**, Numbers of control subjects, subjects with suspected COVID-19, and patients with confirmed mild, regular, severe and critically ill forms of COVID-19 in cohorts 1 and 2. **b**, Numbers of patients that are cured, deceased and with unknown outcome in the two cohorts. **c**, Numbers of chest CT slices of patients with or without COVID-19 pneumonia and cured and deceased cases in the two cohorts. **d**, Statistical comparisons of CFs between subjects with COVID-19 (type I and type II) and controls (*P* < 10^−5^), between type II and type I cases (*P* < 10^−9^), and between deceased and cured cases (*P* < 10^−5^). Two-sided unpaired *t*-test was performed for data following a normal distribution; otherwise a Mann–Whitney *U* test was used. ALG, albumin/globulin ratio; ALB, albumin; ALP, alkaline phosphatase; APTT, activated partial thromboplastin time; AST, aspartate aminotransferase; BUN, urea nitrogen; CA, calcium; CRP, C-reactive protein; DBIL, direct bilirubin; DD, D-dimer; EO, eosinophil count; EOP, eosinophil percentage; GGT, γ-glutamyltransferase; GLB, globulin; GLU, glucose; HSCRP, high-sensitivity C-reactive protein; IL-6, interleukin-6; INR, international normalization ratio; LDH, lactate dehydrogenase; LY, lymphocyte count; LYP, lymphocyte percentage; MOP, monocyte percentage; NE, neutrophil count; NEP, neutrophil percentage; PCT, procalcitonin; PT, prothrombin time; RDWCV, red cell volume distribution width; RDWSD, standard deviation of red cell volume distribution width; TBIL, total bilirubin; and WBC, white blood cell. The full list and details of the CFs are presented in Supplementary Data 1. Further details on the statistical analyses are presented in Supplementary Data 3.

To populate cohorts 1 and 2, we enrolled 1,521 individuals, including 1,126 from Union Hospital (HUST-UH) and 395 from Liyuan hospital (HUST-LH). The data characteristics of cohorts 1 and 2 are shown in Table 1. All patients subjects had CF data, and 1,342 subjects had both CT and CF data. The clinical morbidity outcomes of the 1,521 individuals were classified as (1) 894 patients with confirmed COVID-19 and pneumonia severity ranging from mild (24 cases, 2.7%), regular (596 cases, 66.7%), severe (202 cases, 22.6%), to critically ill (72 cases, 8.1%) forms, (2) 328 COVID-19-negative cases (treated as the control group), and (3) 299 suspected COVID-19 cases, on the basis of the Guidance^16^ (Fig. 1a). Due to the limited data on mild and critically ill forms, we defined mild and regular forms as type I, and severe and critically ill forms as type II. The mortality outcomes of those with confirmed COVID-19 were also counted, including 662 cured cases, 57 deceased cases and 175 cases with unknown outcome (patients transferred to other hospitals) (Fig. 1b).

**Table 1:** The data characteristics of cohort 1 and cohort 2

Type	Cohort 1		Cohort 2	
	No. of patients	No. of CT slices	No. of patients	No. of CT slices
**Morbidity outcome**				
Control	222	54,853	106	27,386
Mild	23	3,246	1	458
Regular	415	92,485	181	45,027
Severe	146	38,583	56	17,549
Critically ill	65	7,901	7	1,010
Suspected	299	75,859	0	0
**Mortality outcome**				
Deceased	53	4,935	4	0
Cured	450	105,636	212	59,362
Unknown	146	31,644	29	4,682

From the original CT images in DICOM format, 364,357 CT slices in JPEG format were exported from 1,342 subjects with chest CT data, including 82,239 slices (22.6%) from 313 control subjects, 3,704 slices (1.0%) from 21 patients with the mild form, 137,512 slices (37.7%) from 543 patients with the regular form, 56,132 slices (15.4%) from 170 patients with the severe form and 8,911 slices (2.4%) from 35 patients with the critically ill form of COVID-19, and 75,859 slices (20.8%) from 260 patients with suspected COVID-19 (Fig. 1c). A total of 164,998 CT slices were obtained from the cured cases and 4,935 CT slices were obtained from the deceased (Fig. 1c).

The CF data were classified into 130 types from 9 categories, including basic information, routine blood test, inflammation test, blood coagulation test, biochemical test, immune cell typing, cytokine profile test, autoimmune test and routine urine test (Supplementary Data 1). The information regarding underlying diseases (Udis) and morbidity outcomes of patients from the two hospitals is presented in Supplementary Data 2. For statistical comparisons of different groups of patients, we first analysed each of the 125 numerical CFs, with the exception of morbidity outcome, mortality outcome, SARS-CoV-2 RNA test, gender and Udis (Supplementary Data 1). In addition to carrying out full statistical tests (Supplementary Data 3), the most significantly different results were visualized (Supplementary Fig. 1). Compared with the controls, 4 types of CFs were significantly higher in patients with COVID-19, including body temperature (average 37.9 °C versus 37.1 °C in controls), GLB, HSCRP and age (average 56.6 yr versus 51.1 yr in controls) (Fig. 1d, Supplementary Fig. 1a and Supplementary Data 3). In patients with COVID-19, 14 CFs were significantly decreased, such as coefficient variation of RDWCV, PCT and ALP (Fig. 1d, Supplementary Fig. 1b and Supplementary Data 3). We also compared type I and II patients. Compared with type I patients, 24 CFs were markedly increased and 14 CFs were markedly decreased in type II patients (Fig. 1d, Supplementary Fig. 1c,d and Supplementary Data 3). Thus, the number of CFs that differed between type II and type I patients was higher than between COVID-19 and control cases, indicating that CF data might be informative for classification of type II and type I patients. Compared with type I patients, age (average 63.6 yr in type II versus 53.1 yr in type I) was significantly higher in type II patients (Fig. 1d and Supplementary Data 3), suggesting an association between age and illness severity, consistent with recent reports^21,22^.

In addition, we compared deceased and cured cases, and observed 16 CFs that were significantly increased and 8 CFs that were significantly decreased (Fig. 1d, Supplementary Fig. 1e,f and Supplementary Data 3), indicating that CFs were informative for predicting mortality outcomes. Further analysis demonstrated that the proportions of patients with Udis were similar among control subjects and patients with COVID-19 (type I and type II) (Supplementary Data 3), suggesting a general susceptibility. Of note, type II cases had a higher proportion of patients with Udis than type I cases, and patients with Udis were also enriched among deceased individuals compared with cured individuals (Supplementary Data 3), revealing a link of Udis with COVID-19 disease severity, consistent with a previous study^23^.

### An integrative resource of COVID-19 pneumonia

Using the two cohorts, we developed iCTCF to maintain and share the corresponding CT images (in both DICOM and JPEG formats), CFs and SARS-CoV-2 laboratory test results. On the resource page (http://ictcf.biocuckoo.cn/Resource.php), multiple options can be selected to search the database, including hospital (HUST-UH and/or HUST-LH), age (<40 yr, 40–60 yr and/or >60 yr), gender (female or male), laboratory-confirmed SARS-CoV-2 status (positive and/or negative), CT evidence (positive, negative and/or not available), and form of COVID-19 (critically ill, severe, regular, mild, suspected and/or control) (Supplementary Fig. 2a). Desired selections can be customized. Clicking the ‘Submit’ button, causes the results to be displayed in a tabular list with information from 20 patients per page (Supplementary Fig. 2a).

For convenience, we also provide an ‘example’ button that can be clicked to automatically load pre-configured selections, followed by the representation of several typical cases (Supplementary Fig. 2b). In this example, we select ‘Patient 4’ to show the annotations in iCTCF. Patient 4 had intermittent fever (a maximum of 38.5 °C), fatigue, shortness of breath and myalgia for 10 d before admission. He coughed occasionally with sputum. On 3 February 2020, the RT–PCR test for SAS-CoV-2 RNA on his throat swab specimens was positive. He was admitted to hospital on 4 February 2020 with finger blood oxygen saturation of 90% in ambient air. This increased to 98% with face mask oxygen support (3 l min^−1^). According to the China Guidance^16^, this patient was diagnosed with a severe form of COVID-19 and regarded as a type II case in iCTCF (Supplementary Fig. 2c).

Clicking on ‘Patient 4’ brings up a display of detailed information on the anonymous patient (Supplementary Fig. 2c). The patient page shows a brief summary of the patient, with five representative CT images (Supplementary Fig. 2c,d). All numerical CFs are provided in a tabular list, and the laboratory-confirmed SARS-CoV-2 status is also shown (Supplementary Fig. 2c,e). Consistent with the brief clinical summary described above, the age of the patient (73 yr), gender (male), body temperature (38.5 °C), positive SARS-CoV-2 infection status, and Udis (aorta calcification) are presented. The patient’s CT examination suggests possible bilateral viral pneumonia. The patient’s tabular list contains 82 numerical CFs, including but not limited to the decrease of EO, LY, EOP and LYP, and increased levels of neutrophil percentage (NEP), erythrocyte sedimentation rate (ESR) and CRP (Supplementary Fig. 2e).

### Development of HUST-19 for predicting clinical outcomes of COVID-19 patients

**Fig. 2:**
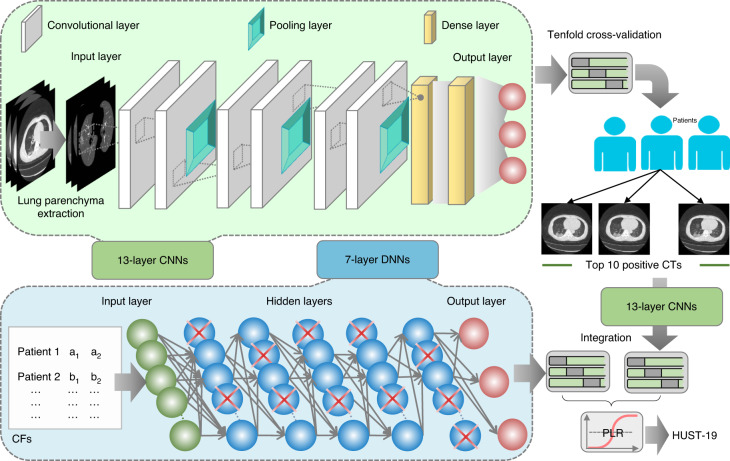
The hybrid learning architecture of HUST-19. HUST-19 includes a 13-layer CNN framework for predicting individual CT slices, a second 13-layer CNN framework to transform individual slice-based prediction into patient-based prediction of clinical outcomes, a 7-layer DNN framework to predict clinical outcomes of patients with COVID-19 from CFs, and the PLR algorithm used for integration of CT- and CF-based results to predict morbidity or mortality outcomes.

To exemplify the usefulness of iCTCF, we developed HUST-19, which integrates the CT slice and CF datasets to predict clinical outcomes (both morbidity and mortality outcomes) of patients with COVID-19 pneumonia. HUST-19 comprises four steps, including classification of individual CT slices, CT-based prediction of clinical outcomes, CF-based prediction of clinical outcomes, and integration of CT- and CF-based predictions (Fig. 2).

First, we classified individual CT slices into three types: (1) non-informative CT (NiCT) images, in which lung parenchyma was not captured for any judgement; (2) positive CT (pCT) images, in which imaging features associated with COVID-19 pneumonia could be unambiguously discerned; and (3) negative CT (nCT) images, in which imaging features in both lungs were irrelevant to COVID-19 pneumonia. To enable slice-based prediction, we implemented a deep learning framework based on the architecture of VGG-16, a classic convolutional neural network (CNN) framework for image recognition^24^. The original VGG-16 contained 16 weight layers including 13 convolutional and 3 fully connected (dense) layers, but too many parameters needed fine tuning. To reduce the model complexity and enable faster training, we retained only 6 convolutional and 2 dense layers. The simplified CNNs contained 13 layers, including one input layer, 3 sets of dual convolutional and pooling layers (3 × 3), 2 dense layers, and one output layer (Fig. 2). The 13-layer CNNs were used to classify individual CT slices into three types: NiCT, pCT and nCT. Second, an additional framework of 13-layer CNNs was implemented to transform the individual CT slice-based prediction into the patient-based prediction (Fig. 2). For each patient, the ten most probable pCT images were retained as representative images, which were input into the secondary 13-layer CNNs to predict clinical outcomes. Third, the CF-based prediction of patients was implemented in a framework of seven-layer deep neural networks (DNNs), including one input layer, five dense layers, and one output layer (Fig. 2). In contrast to CNNs, DNNs did not have convolutional and pooling layers. Finally, the predictions using CT slices or CFs were integrated through the penalized logistic regression (PLR) algorithm to output final predictions on morbidity or mortality outcomes of patients. All pre-configured parameters in CNN and DNN frameworks are shown in Supplementary Data 4.

### The prediction accuracy of HUST-19

**Fig. 3:**
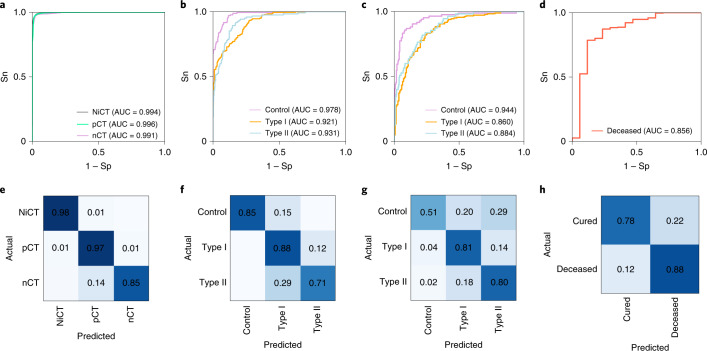
The performance evaluation of HUST-19 based on tenfold cross-validations. **a**, The slice-based prediction of NiCT, pCT and nCT images. **b**,**c**, The integration of CT and CF data for predicting morbidity outcomes in cohort 1 (207 controls, 384 type I patients and 149 type II patients having both CT and CF data) (**b**) and cohort 2 (106 controls, 180 type I patients and 56 type II patients having both CT and CF data) (**c**). **d**, The integration of CT and CF data for predicting mortality outcomes on the merged cohort (594 cured and 19 deceased cases having both CT and CF data). **e**–**h**, Corresponding confusion matrices for the four types of predictions in **a**–**d**, respectively, are derived from the tenfold cross-validations under the sensitive threshold. Further details on the performance evaluation are provided in Supplementary Data 4.

Details on the performance evaluation of HUST-19 are presented in Tables 2 and 3. First, using 19,685 manually labelled CT slices, tenfold cross-validations were conducted to evaluate the performance of the slice-based prediction. From the receiver operating characteristic (ROC) curve, HUST-19 achieved an AUC value of 0.994 in distinguishing pCT and nCT images from NiCT images, and an AUC value of 0.991 in predicting pCT images (Fig. 3a and Table 2). To train models to predict morbidity outcomes, we used 197,068 CT slices and 127 types of CF data from 222 controls, 438 type I patients and 211 type II patients in cohort 1. The integration of CT and CF data achieved AUC values of 0.978, 0.921 and 0.931 in predicting controls, type I and type II patients, respectively (Fig. 3b and Table 2). We also used the cohort 2 as a validation dataset to test HUST-19, which still exhibited promising accuracy (Fig. 3c and Table 3). In cohort 2, there were 91,430 CT slices and CF data from 106 controls, 182 type I patients and 63 type II patients. To train models to predict mortality outcomes, cohorts 1 and 2 were merged with 169,933 CT slices and CF data from 662 cured and 57 deceased cases, due to data limitation. From the results, we observed that the mortality outcomes could be accurately predicted, with an AUC value of 0.856 (Fig. 3d and Table 2).

**Table 2:** Details on the performance evaluation of HUST-19 for the prediction of individual CT slices, morbidity outcomes, and mortality outcomes

Prediction	Type	AUC	Sn (%)	Sp (%)	Ac (%)	PPV (%)	NPV (%)	MCC
Prediction of individual CT slices	NiCT	0.994	98.40%	99.64%	99.42%	99.12%	99.55%	0.9861
	pCT	0.996	97.00%	90.68%	91.97%	72.74%	99.16%	0.7940
	nCT	0.991	85.47%	99.12%	92.38%	99.00%	87.25%	0.8557
**Prediction of morbidity outcomes**								
CT based	Control	0.919	51.99%	98.01%	84.66%	91.46%	83.32%	0.6115
	Type I	0.804	94.70%	39.17%	67.76%	62.29%	87.45%	0.4105
	Type II	0.838	19.98%	98.33%	83.05%	74.39%	83.53%	0.3257
CF based	Control	0.882	49.95%	96.75%	84.55%	84.43%	84.57%	0.5677
	Type I	0.856	92.58%	47.56%	68.92%	61.82%	86.68%	0.4385
	Type II	0.879	44.96%	98.04%	84.27%	88.94%	83.56%	0.5583
HUST-19	Control	0.978	85.01%	99.80%	95.31%	99.46%	93.86%	0.8897
	Type I	0.921	87.82%	79.20%	83.34%	79.55%	87.59%	0.6708
	Type II	0.931	70.86%	92.67%	87.94%	72.79%	91.99%	0.6415
**Prediction of mortality outcomes**								
CT based		0.808	76.47%	76.40%	76.41%	13.40%	98.55%	0.5000
CF based		0.822	81.13%	70.32%	71.49%	24.86%	96.86%	0.4994
HUST-19		0.856	88.24%	78.26%	78.73%	16.67%	99.26%	0.5236

The Sn, Sp, accuracy (Ac), positive predictive value (PPV), negative predictive value (NPV) and Matthews correlation coefficient (MCC) were calculated from the tenfold cross-validations.

**Table 3:** Details on the performance evaluation of HUST-19 for the prediction of morbidity outcomes using data from cohort 2

Prediction	Type	AUC	Sn (%)	Sp (%)	Ac (%)	PPV (%)	NPV (%)	MCC
CT based	Control	0.895	53.57%	94.47%	83.70%	77.59%	85.06%	0.5486
	Type I	0.775	86.67%	50.36%	70.85%	69.33%	74.47%	0.4027
	Type II	0.832	32.73%	95.08%	84.33%	58.06%	87.15%	0.3546
CF based	Control	0.888	54.72%	99.59%	86.04%	98.31%	83.56%	0.6668
	Type I	0.834	72.53%	78.70%	75.50%	78.57%	72.68%	0.5124
	Type II	0.845	71.63%	78.82%	77.49%	42.45%	92.65%	0.4200
HUST-19	Control	0.944	51.19%	98.30%	85.89%	91.49%	84.93%	0.6150
	Type I	0.860	80.56%	76.26%	78.68%	81.46%	75.18%	0.5673
	Type II	0.884	80.00%	78.41%	78.68%	43.56%	94.95%	0.4743

The Sn, Sp, Ac, PPV, NPV and MCC values were directly calculated.

In HUST-19, we selected a sensitive threshold to maximize the ability to correctly predict the clinical outcomes of true COVID-19 patients. Under this threshold, confusion matrices were generated to visualize the agreement between actual and predicted results (Fig. 3e–h). For the CT slice-based prediction, it was found that NiCT, pCT and nCT slices could be correctly recognized with high accuracy (Fig. 3e). For predicting morbidity outcomes, different types of cases could be clearly distinguished for both cohort 1 (Fig. 3f) and cohort 2 (Fig. 3g). The deceased and cured cases could also be accurately separated (Fig. 3h). In addition, ROC curves and confusion matrices were plotted for exclusively using CT or CF data to predict morbidity or mortality outcomes (Supplementary Fig. 3). The results supported the proposed combining of CT and CF data to achieve a higher accuracy for predicting the clinical outcomes (Tables 2, 3 and Supplementary Fig. 3).

### Computational annotations of suspected cases

**Fig. 4:**
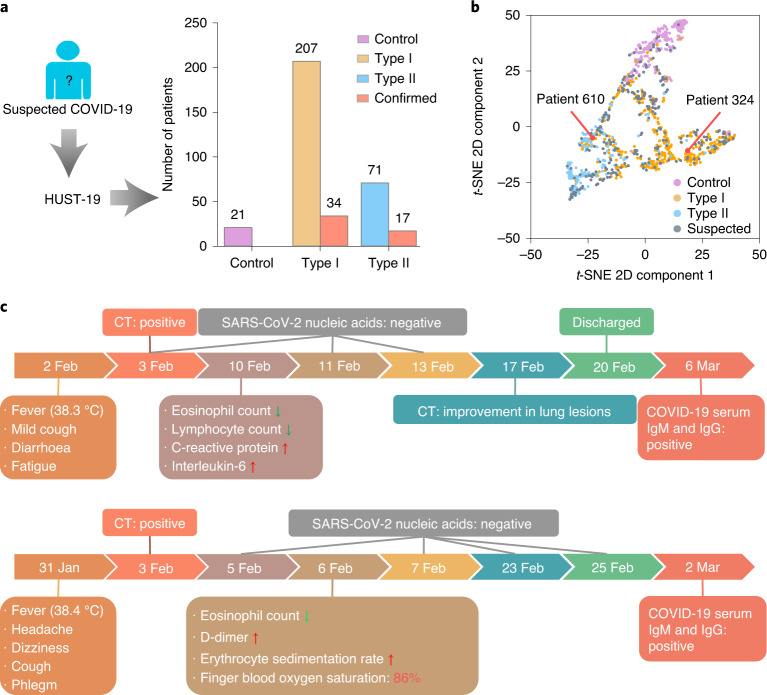
Prediction of potential morbidity outcomes of 299 suspected cases without laboratory confirmation of SARS-CoV-2 status at the time of enrolment. **a**, HUST-19 was used to predict whether 299 suspected cases were COVID-19 negative, or type I or type II cases (Supplementary Data 4). **b**, *t*-SNE analysis of the classification efficiency of HUST-19 for the predictions described in **a**. **c**, Schematics showing the clinical courses of two suspected cases of COVID-19, patient 324 and patient 610, who were predicted by HUST-19 to correspond to type I and type II cases, respectively. Jan, January; Feb, February; Mar, March.

In iCTCF, there were 299 suspected cases without definitive laboratory confirmation of SARS-CoV-2 at the time of enrolment. We used HUST-19 to predict 21 of these to be COVID-19 negative, 207 to be type I cases and 71 to be type II cases (Fig. 4a). Among the predictions, 51 suspected cases were since confirmed as COVID-19-positive cases during this study. For each patient, the six intermediate scores generated from CT image-based and CF-based predictions were retrieved and analysed using *t*-distributed stochastic neighbour embedding (*t*-SNE) in a 2D plot. The *t*-SNE results demonstrated that the suspected cases were dispersed among the three types, and the predicted type I and II cases were closely grouped with confirmed COVID-19-positive cases (Fig. 4b). For example, patients 324 and 610 were predicted to correspond to type I and II cases, respectively (Fig. 4b).

Patient 324 (female, 34 yr old) was admitted to HUST-UH on 9 February 2020, because of “fever for seven days” and ground-glass opacity in the left lower lung suggested by CT imaging (Fig. 4c). Her maximum recorded body temperature was 38.3 °C, and was accompanied by mild cough, diarrhea and fatigue. On 10 February 2020 (day 1 after admission), blood biochemical examination showed eosinopenia, lymphopenia, elevated HSCRP and increased IL-6 concentration. However, the results of three laboratory tests for SARS-CoV-2 RNA, on 3 February (at the clinic), 11 February (day 2 after admission) and 13 February (day 4 after admission), were negative. Since she was afebrile from 12 February (day 3 after admission) and CT indicated a substantial improvement in lung lesions on 17 February (day 8 after admission), this patient was discharged with the diagnosis of “suspected COVID-19 regular form” on 20 February (day 11 after admission). On the basis of the CT and CF data, the HUST-19 model predicted this patient to be a type I case of COVID-19. Her COVID-19 infection was subsequently verified by positive tests for COVID-19 serum immunoglobulin M (IgM) and immunoglobulin G (IgG) when she returned to the hospital for a follow-up examination on 6 March (day 15 after discharge). Thus, her diagnosis was eventually corrected to be “COVID-19 regular form” (Fig. 4c).

Another example was patient 610 (female, 73 yr old) (Fig. 4c), who was admitted to HUST-UH on 6 February 2020 because of “fever, headache, and dizziness for a week” and multiple GGO lesions in CT images of both lungs. Her body temperature was 38.4 °C, accompanied by shortness of breath, coughing and phlegm. She had hypertension that was under control. On admission, her laboratory tests showed eosinopenia, an elevated level of DD and an increased ESR. During hospitalization, the patient had dyspnea with finger blood oxygen saturation below 93% in ambient conditions, which was corrected with face mask oxygen support (10 min l^−1^, 86%). However, the results of four laboratory tests for SARS-CoV-2 RNA, on 5 February (day 1 before admission), 7 February (day 1 after admission), 23 February (day 17 after admission) and 25 February (day 19 after admission), were negative. She was eventually confirmed to have COVID-19 by positive results for serum SARS-CoV-2 IgM and IgG on 2 March (day 25 after admission). Thus, she was diagnosis with a severe case of COVID-19 (Fig. 4c). On the basis of her CT and CF data, HUST-19 also accurately classified this case as a type II COVID-19 case, without knowledge of her SARS-CoV-2 infection status (Fig. 4b). In sum, our annotation of suspected cases suggests that HUST-19 can be a helpful tool for identifying patients with COVID-19, and can quickly provide useful information towards further diagnosis and treatment of the disease.

## Discussion

Over the last few months, the outbreak of COVID-19 pneumonia has affected nearly 200 countries and regions, and endangered millions of lives. Since the early stage of the outbreak, the local hospitals in Wuhan have been accepting patients with COVID-19 and have accumulated substantial amounts of first-hand COVID-19-related CT and clinical data. We believe that timely and consistent curation of CT imaging, clinical and laboratory information on COVID-19 is important to help understand the scope of disease impact, the spectrum of pathophysiological features, and the risk factors for progressive worsening, and to provide a reliable resource for diagnostic modelling and retrospective analysis that can inform screening, triage and diagnosis, and facilitate risk management and treatment efforts to effectively combat this disease. Further, detailed information provided at large scale in real time can be critical for revealing robust clinical findings, deciding where to prioritize research or therapeutic efforts, and for building consensus among clinicians.

It is rare for large datasets to be freely available for sharing during disease outbreaks. Several published papers and preprints have reported the use of cutting-edge artificial intelligence for predicting COVID-19 diagnosis on the basis of CT imaging and relevant clinical information^25–35^. Although these studies have highlighted the potential value of machine learning in diagnosis of COVID-19, the datasets used to build their algorithms were relatively small and lacked free public access. In a recent study, a deep learning-based method was developed to diagnose patients with or without COVID-19 pneumonia, using mainly CT imaging data^25^. The mortality outcomes could not be predicted, and the original DICOM files of the dataset used in the study were not provided. Only segmented slices of lung parenchyma in JPEG format were presented. By contrast, our iCTCF database has an open, publicly accessible computational infrastructure. It systemically integrates CT images and CFs from patients with or without COVID-19 pneumonia. iCTCF is a comprehensive repository for COVID-19 pneumonia, and is, to our knowledge, the largest CT imaging and CF characteristics database of COVID-19 to date.

Given the rapid surge in the number of cases of COVID-19 globally, patients has been overloading local medical systems in many regions. A key to control this epidemic is to diagnose COVID-19 as early as possible, in order to apply timely medical interventions, such as isolation or treatment, with a goal of reducing cross-infection and blocking illness progression in individual patients. To facilitate COVID-19 diagnosis and demonstrate the usefulness of iCTCF, we developed an engineering framework of HUST-19 using CT slices and CF data, and achieved AUC values of 0.921, 0.931 and 0.856 in predicting type I, type II and deceased cases, respectively (Fig. 3). In addition, we found that HUST-19 achieved a much higher accuracy than using either CT data or CF data (Supplementary Fig. 3).

In addition to HUST-19, we also implemented two additional open-source CNN frameworks using our CT data, Inception Net V3^36^ and ChexNet^37^, for predicting morbidity and mortality outcomes, respectively. The original architecture of Inception Net V3 contains 11 inception modules, which were truncated to only 3 inception modules and a grid-size reduction module to accurately predict COVID-19 using chest X-ray images^38^. ChexNet was developed based on a 121-layer dense convolutional network (DenseNet-121)^39^ to predict 14 types of thoracic diseases, including pneumonia from chest X-ray images^37^. For predicting morbidity outcomes, we re-trained the Inception Net V3 and ChexNet models using cohort 1; their performance values on cohort 1 and cohort 2 are presented in Supplementary Fig. 4a–d). The same dataset used in HUST-19 was adopted to train the Inception Net V3 and ChexNet models for predicting mortality outcomes. Tenfold cross-validations were performed to evaluate their accuracy (Supplementary Fig. 4e,f). The results indicated that the Inception Net V3 and ChexNet models achieved similar accuracies to HUST-19 for predicting morbidity and mortality outcomes (Supplementary Figs. 3a,c,e and 4). These results demonstrated that CNN-based models could accurately predict COVID-19 using CT data.

### Outlook

We hope to continuously archive relevant COVID-19 information into the iCTCF system for sharing. We compared the China Guidance^16^ and the US Guidelines^40^ for COVID-19, and found that the definitions of mild, regular, severe and critically ill forms of COVID-19 patients in the China Guidance^16^ are consistent with the ‘mild illness’, ‘moderate illness’, ‘severe illness’, and ‘critical illness’ forms defined in the US Guidelines^40^, respectively. Thus, iCTCF and HUST-19 should be applicable beyond China. With abundant and reliable information, the iCTCF database can be a valuable resource for improving the diagnosis and clinical management of COVID-19.

## Methods

### COVID-19 case definitions and clinical classifications

Patients were diagnosed as suspected cases of COVID-19 or confirmed cases with mild, regular, severe and critically ill forms as described^16^. Due to limited data regarding mild forms (24 cases) and critically ill forms (72 cases) in cohorts 1 and 2, morbidity outcomes were defined as mild or regular (type I) and severe or critically ill (type II) forms, depending on the severity of the disease.

Specifically, suspected cases were defined if they met the criteria: any of the following epidemiological history plus any two of following clinical manifestations, or all the three clinical manifestations without clear epidemiological history. The epidemiological history included: (1) recent travel history in and around Wuhan, or other communities with reported cases within 14 d prior to the disease onset; (2) contact history with COVID-19-infected case(s) (positive RNA test) within 14 d prior to the disease onset; (3) contact history with patient(s) having fever or respiratory symptoms from Wuhan or surrounding areas, or reported communities within 14 d prior to the disease onset; (4) a cluster of the disease onset. The clinical manifestations included: (1) fever and/or respiratory symptoms; (2) chest CT imaging evidence showing signs of COVID-19 pneumonia, including the appearance of multiple small patchy GGOs, interstitial changes and peripheral lung abnormality at the early stage, rapid progression to multiple focal or diffuse bilateral GGOs, and consolidations in severe cases; (3) laboratory findings of normal or decreased number of leukocytes or lymphopenia at the early stage of disease onset.

If suspected cases had definitive positive evidence of SARS-CoV-2 RNA (RT–PCR positive for specimens such as throat swabs), they were diagnosed as confirmed COVID-19. According to the Guidance^16^, the morbidity outcomes of the confirmed COVID-19 cases were clinically classified as the following four forms on the basis of illness severity: (1) mild form: mild clinical symptoms without chest CT imaging signs of viral pneumonia; (2) regular form: fever and respiratory symptoms with chest CT imaging signs of viral pneumonia; (3) severe form: should meet any of the following criteria, (i) anhelation (respiratory rate ≥ 30 breaths per min) or (ii) finger blood oxygen saturation ≤ 93% in ambient condition, (iii) arterial partial pressure of oxygen (PaO_2_)/fraction of inspiration oxygen (FiO_2_) ≤ 300 mmHg (1 mmHg = 0.133 kPa); adjusted in high-altitude areas (>1,000 m above sea level) using a formula PaO_2_/FiO_2_ × barometric pressure (mmHg)/760, and (iv) more than 50% lesions in chest CT imaging are clearly developed within 24 to 48 h; (4) critically ill form: should meet any of the following criteria: (i) respiratory failure requiring mechanical ventilation, (ii) shock, and (iii) concurrently having other organ failure that needs intensive care unit treatment.

According to the COVID-19 Treatment Guidelines released by the US National Institutes of Health (updated on 30 July 2020)^40^, COVID-19 patients are categorized into five forms, including asymptomatic or presymptomatic infection, mild illness, moderate illness, severe illness, and critical illness. Except for the suspected form in China and the asymptomatic or presymptomatic infection form in the United States, the definitions of mild, regular, severe and critically ill forms in the China Guidance are highly similar to mild illness, moderate illness, severe illness, and critical illness forms, respectively, in the US Guidelines.

### Data collection and preparation

The collection, use, and retrospective analysis of chest CT images, CFs and SARS-CoV-2 RT–PCR results from patients were approved by the institutional ethics committees of HUST-UH (IRB ID: [2020] IEC (A001)) and HUST-LH (IRB ID: [2020] IEC (A001)). Informed patient consent was waived by the ethics committees due to the COVID-19 emergency. For all enrolled patients, the first sets of CT and CF data after admission were collected. The daily medical records of cases from HUST-UH and HUST-LH were manually checked and confirmed by three attending physicians (J. Zhang, a senior respiratory physician with more than 10 yr experience; Q. You, a senior physician with more than 10 yr experience in infectious disease; and J. Wang, a senior physician with more than 10 yr experience in infectious disease), and the medical records of HUST-LH were checked and confirmed by two attending physicians (Y.Z., a senior respiratory and critical care physician with more than 30 yr experience, and H. Peng, a senior respiratory and critical care physician with more than 20 yr experience). Clinical classifications (that is, morbidity outcomes) of COVID-19 for each patient were determined and confirmed by these same physicians according to the Guidance^16^. Any ambiguous or inconsistent records were resolved by discussion with these attending physicians.

In cohort 1, the data were from (1) patients receiving PCR testing who were hospitalized between 25 January and 20 February 2020 at HUST-UH and HUST-LH; (2) patients admitted to HUST-UH between 14 November and 30 November 2019, who were diagnosed with community-acquired pneumonia; (3) healthy individuals having a routine physical check-up. The 1,170 patients included 775 patients from HUST-UH and 395 patients from HUST-LH. There were 222 control cases comprising 112 patients with community-acquired pneumonia, 14 healthy individuals and 96 patients who tested negative for SARS-CoV-2 RNA and for whom CT imaging showed no signs of COVID-19 infection. The 649 laboratory-confirmed COVID-19 patients comprised 23 mild, 415 regular, 146 severe and 65 critically ill cases. The remaining 299 subjects were suspected cases. Among these 1,170 individuals, 1,000 had CT images (a total of 272,927 CT slices). Among the confirmed cases, there were 450 cured cases and 146 cases with unknown outcome (patients transferred to other hospitals during hospitalization). The remaining 53 deceased subjects included 39 who had the critically ill form and 14 who had the severe form. Because of their severe illness, only 17 of the deceased cases had CT examinations.

To further evaluate the accuracy of HUST-19, we prepared cohort 2 from (1) patients who given RT–PCR tests and were admitted between 14 February and 29 February 2020 to HUST-UH; (2) patients admitted to HUST-UH between 20 August and 30 November 2019 and diagnosed with community-acquired pneumonia. These 351 patients included 245 patients with laboratory-confirmed COVID-19 and 106 control cases. Among the morbidity outcomes were 1 mild, 181 regular, 56 severe and 7 critically ill cases among the confirmed cases. Among the mortality outcomes, there were 212 cured cases and 29 case with unknown outcome. The remaining 4 deceased cases included 3 from the critically ill form and 1 from the severe form. The data in cohort 2 was from the same hospitals as the data from cohort 1. Thus, cohort 2 was not a fully independent dataset. Cohort 2 was taken as a validation dataset and not used for model training.

### Clinical examinations procedure

Patients received nine classes of clinical examinations, including basic information, routine blood tests, inflammation tests, blood coagulation tests, biochemical tests, immune cell typing, cytokine profile tests, autoimmune tests and routine urine tests. These tests were performed in the clinical laboratory departments of HUST-UH and HUST-LH, and the results were collectively denoted as CFs. The basic information included morbidity outcomes, mortality outcomes, SARS-CoV-2 RNA tests, age, gender, body temperature (°C), and Udis, which were taken from patients’ medical records.

At HUST-UH, routine blood tests, such as haemoglobin (HGB), were carried out by a Sysmex XE-5000 automatic blood analyser (Sysmex). To test for inflammation, ESR was detected by a Monitor 100 (Vital Diagnostics) and CRP was tested by a BN II (Siemens). Blood coagulation tests were carried out by a STA-R Evolution (Stago). Biochemical tests were done by an AU5800 (Beckman Coulter). B-type brain natriuretic peptide precursor (BNP) was detected by an Architect i2000 (Abbott). Quantification and typing of immune cells was conducted using flow cytometry (Cytomics FC 500, Beckman Coulter). The cytokine profile tests, such as detection of IL-2, IL-4, IL-6, IL-8, IL-10, TNF and IFN-γ, were also quantitatively determined by a Cytomics FC 500 (Beckman Coulter). Autoimmune tests of complement proteins (C1q, C3 and C4) and immunoglobulins (IgA, IgM and IgG) were conducted in an IMMAGE 800 (Beckman Coulter), while anti-streptolysin O (ASO) and rheumatoid factor (RF) were tested in a BN II (Siemens). Routine urine tests were performed using a Sysmex UF-1000i (Sysmex).

At HUST-LH, routine blood tests were performed by a BC5390 automatic blood cell analyser (Mindray). To test for inflammation, CRP was tested by a BC5390 automatic blood cell analyser (Mindray). PCT was tested by a Pylon immunoassay (ET Healthcare). HSCRP was tested by an AU5800 (Beckman Coulter). Blood coagulation tests were carried out by a Sysmex CS5100 automatic blood coagulation analyser (Sysmex). For biochemical tests and autoimmune tests, an AU5800 (Beckman Coulter) was used. Fungal (1,3)-β-d-glucan (FDG) was tested with an LKM series dynamic test tube detector (Labkinetics). BNP was analysed using a Cobas e601 full-automatic electrochemical luminescence immunoassay system (Roche). Routine urine tests were performed by an FUS-2000 fully automatic urine analysis workstation (DIRUI).

### Chest CT image acquisitions

At HUST-UH, all patients underwent CT examinations in the supine position on one of the three CT systems: Somatom Definition AS+ (Siemens Healthineers), Discovery 750HD (GE Medical Systems) or Toshiba Activion 16 (Toshiba). The scanning range was set from the thoracic inlet to the diaphragm. The scan parameters were 128 × 0.6 mm or 64 × 0.6 collimation, 120 kV tube voltage and 350 × 350 mm field of view. All datasets were reconstructed with a slice thickness of 1.5–2 mm and an increment of 1.5–2 mm. Due to the excessively large number of patients during the outbreak, CT images were frequently reconstructed with 5 mm layer thickness and 5 mm layer spacing for a considerable proportion of patients to enable a faster examination.

At HUST-LH, patient chest CT scans were performed with a uCT510 spiral CT scanner (United Imaging). The scanning range was set from the thoracic inlet to the diaphragm. The scan parameters were 32 × 0.6 mm collimation, 120 kV tube voltage and 350 × 350 mm field of view. All patients were in the supine position, and the patients were trained to breathe before the scan. During scanning, patients were asked to hold their breath. The scan ranged from the tip of the lungs to the lower edge of the costal angle. The original data were reconstructed into an image with 1.5 mm layer thickness and 1.2 mm layer spacing.

From the two hospitals, original CT images in DICOM format were obtained for all enrolled cases. To ensure patients’ anonymity, a script was written in Python 3.7 to remove personal information and CT examination date from DICOM files.

### CT slice labelling and interpretation

For training individual slice-based models in HUST-19, we manually labelled 19,685 CT slices exported from DICOM images after removing personal information for 61 COVID-19 patients and 43 control cases. During labelling clinical or laboratory findings were not accessed. Individual CT slices in JPEG format from cases from HUST-UH were labelled and interpreted by two radiologists (H.S., a senior thoracic radiologist with more than 30 yr experience and Y.C., a radiologist with 5 yr experience in interpreting chest CT images). The CT slices from cases from HUST-LH were labelled and interpreted by two radiologists (H.Z., a senior radiologist with 15 yr experience and H. J. Zhang, a radiologist with 5 yr experience in interpreting chest CT images). The radiologists independently labelled CT slices, and resolved any disagreements through discussion to achieve consensus and interpretation of CT imaging features. In total, we obtained 5,705 NiCT, 4,001 pCT and 9,979 nCT slices.

### Laboratory confirmation of COVID-19 pneumonia

The aetiological confirmation of SARS-CoV-2 infection was done by RT–PCR amplification of the *ORF1ab* and *N* genes of SARS-CoV-2 (BioGerm) from throat or nasopharyngeal swab specimens from patients. The primers for amplification and detection of the *ORF1ab* were: forward, 5′-CCCTGTGGGTTTTACACTTAA-3′; reverse, 5′-ACGATTGTGCATCAGCTGA-3′; and fluorescent probe, 5′-FAM-CCGTCTGCGGTATGTGGAAAGGTTATGC-BHQ1-3′. The primers for amplification and detection of the *N* gene were: forward, 5′-GGGGAACTTCTCCTGCTAGAAT-3′; reverse, 5′-CAGACATTTTGCTCTCAAGCTG-3′; and fluorescence probe, 5′-FAM-TTGCTGCTGCTTGACAGATT-TAMRA-3′. A cycle threshold value less than 35 (or between 35 and 38 twice) was defined as positive.

### Performance evaluation

To evaluate the accuracy of HUST-19, true positive (TP), true negative (TN), false positive (FP) and false negative (FN) values were counted. Then, we calculated six measurements, Sn, Sp, Ac, PPV, NPV and MCC, as below:

1 $$\mathrm{Sn} = \frac{\mathrm{TP}}{{\mathrm{TP} + \mathrm{FN}}}$$

2 $$\mathrm{Sp} = \frac{{\mathrm{TN}}}{{\mathrm{TN} + \mathrm{FP}}}$$

3 $$\mathrm{Ac} = \frac{{\mathrm{TP} + \mathrm{TN}}}{{\mathrm{TP} + \mathrm{FP} + \mathrm{TN} + \mathrm{FN}}}$$

4 $$\mathrm{PPV} = \frac{{\mathrm{TP}}}{{\mathrm{TP} + \mathrm{FP}}}$$

5 $$\mathrm{NPV} = \frac{{\mathrm{TN}}}{{\mathrm{TN} + \mathrm{FN}}}$$

6 $$\begin{array}{l}\mathrm{MCC} = \\ \frac{{\left( {\mathrm{TP} \times \mathrm{TN}} \right) - \left( {\mathrm{FN} \times \mathrm{FP}} \right)}}{{\sqrt {\left( {\mathrm{TP} + \mathrm{FN}} \right) \times \left( {\mathrm{TN} + \mathrm{FP}} \right) \times \left( {\mathrm{TP} + \mathrm{FP}} \right) \times \left( {\mathrm{TN} + \mathrm{FN}} \right)} }}\end{array}$$

For each method, the tenfold cross-validation was performed ten times, and average Sn, Sp, Ac, PPV, NPV and MCC values were calculated. The ROC curve was plotted based on final Sn and 1 − Sp scores, and the AUC value was computed. For the computational annotation of suspected cases, *t*-SNE analysis was implemented in Scikit-learn 0.21.2 (https://scikit-learn.org/stable/), a package for data mining and analysis.

### Chest CT slice pre-processing

To extract the lung parenchyma from chest CT slices, we implemented an integrative pipeline for CT slice pre-processing. First, the adaptive threshold segmentation algorithm was applied to convert greyscale CT slices to binary CT slices. Then, we removed the noise of CT slices by only keeping the largest connected architecture, the human body profile. After this manipulation, the background of a CT slice was black, and the black lung parenchyma was surrounded by the white human body profile. To retain only the black lung parenchymal area, the flood fill algorithm was used to fill the CT slice background with white, starting from the border of the CT slices. Finally, the lung parenchyma in the original CT slice was extracted. For non-square CT slices, we cropped them into square slices before pre-processing. To rapidly process and analyse such a large dataset and unify the size of CT slices, we rescaled all CT slices to 200 × 200 pixels and effectively avoided distortion by using bilinear interpolation. The OpenCV 3.4.2 (https://opencv.org/) and Scikit-image 0.15.0 (https://scikit-image.org/) computer vision libraries were adopted for CT slice pre-processing^41^.

### The 13-layer CNNs

We used 2 sets of 13-layer CNNs for CT slice-based and patient-based predictions, respectively. In each CNN framework, there was one input layer, three sets of dual convolutional and pooling layers, two dense layers and one output layer. In the 11 hidden layers, neurons were the basic computation units, and both internal feature coding and computational outcome were connected and propagated by neurons inside each layer. The convolutional layers were used for feature extraction and presentation, and a widely used rectified linear unit (ReLU) function was adopted to activate the outcome of a neuron and defined as below:

7 $${\mathrm{ReLU}}\left( x \right) = \left\{ {\begin{array}{*{20}{c}} {x,x \ge 0} \\ {0,x < 0} \end{array}} \right.$$

Where *x* was the weighted sum of a neuron.

In the pooling layers, feature selection and information filtering were performed by the max-pooling strategy. The last two hidden layers were dense layers for generating prediction outcomes. To prevent overfitting that frequently occurs in deep learning algorithms, we used a simple dropout method to randomly select a number of nodes from the two dense layers and set their corresponding scores to 0 if the average Ac value increased. In the output layer, three softmax nodes were set to separately calculate three scores for an input CT slice shown as below:

8 $${\mathrm{Score}}\left( {y_i} \right) = \frac{{e^{y_i}}}{{\mathop {\sum }\nolimits_{i = 1}^k e^{y_i}}}$$

Where *y*_*i*_ was the input of *i*th softmax node derived from the dense layer and *k* was the number of softmax nodes. In the CNN model for the slice-based prediction, the Score(*y*_*i*_) was a value in the range 0–1 representing the probability of a CT slice classified as a NiCT, pCT or nCT slice. For the patient-based prediction of morbidity outcomes, the Score(*y*_*i*_) was a value in the range 0–1 to reflecting the probability of a patient being a control case, a type I patient, or a type II patient. For predicting mortality outcomes, one softmax node was adopted in the output layer, which produced a value between 0 and 1 to denote the mortality probability.

### Normalization of CF data and the seven-layer DNNs

For each patient, each CF was given a diagnosed value *f*, which was normalized as below:

9 $$F = \frac{{f - \mathrm{Min}}}{{\mathrm{Max} - \mathrm{Min}}}$$

Where *F* was the normalized value of *f*, and the normal range of the CF was Min to Max. If *f* was an unavailable value, we set *F* to 0.5. For the two CFs gender and Udis, we used 0 or 1 to encode males or females, respectively, and adopted 0 and 1 to encode patients with and without Udis, respectively.

To enable the prediction of clinical outcomes of patients based on normalized CFs, we used seven-layer DNNs, including one input layer, five dense layers and one output layer. Again, to avoid overfitting, the dropout method was used by randomly dropping nodes from the five hidden layers if the average Ac value increased. In the first step, the input layer received numerical values of CFs for each patient. The five hidden layers were mainly adopted for feature extraction and representation. The ReLU activation function was used to transform data for each node. For predicting morbidity outcomes, the output layer contained three softmax neurons to separately calculate 3 values ranging from 0 to 1 for each patient. For predicting mortality outcomes, one softmax node was used to calculate the mortality probability.

### The PLR algorithm

The integration of predictions from CT slices and CFs were performed by the PLR algorithm, which was implemented in Python 3.7 with Scikit-learn 0.21.2. For each patient, CNN models and DNN models were individually used to calculate three scores for morbidity outcomes and one score for mortality outcomes. Then, the 6 or 2 intermediate values were taken as secondary features, and the weight score of each value was initially set to 1. The ridge regression (L2 regularization) penalty was adopted to optimize the weight scores if the average Ac value increased. Finally, the PLR model calculated three scores for predicting morbidity outcomes and one score for predicting mortality outcomes.

### Model training and parameter optimization

To train the 13-layer CNN models for individual CT slice-based prediction, we randomly generated a training dataset and a testing dataset with a size ratio of approximately 9:1, in which the labelled NiCT, pCT and nCT slices were proportionally distributed. We further randomly split the training dataset into ten parts, of which nine were used for model training. Then, we used the remaining part to calculate the average Ac value for predicting the three types of CT images, and the process of parameter optimization was stopped when the Ac value was no longer increased. The randomization and parameter optimization on the training dataset was performed ten times, and the model with the highest Ac value was retained. Using the determined parameters, the final model was trained on the full training dataset. The testing dataset was not used for training, and was only used to count TP, TN, FP and FN values and calculate the performance measurements. A similar tenfold cross-validation was also adopted for the CT-based prediction of morbidity or mortality outcomes, the CF-based prediction of morbidity or mortality outcomes, and the integration of predictions from CT images and CFs.

For model training, we used a computer with an Intel Core i7-6700K 4.00 GHz central processing unit, 32 GB of RAM and a NVIDIA GeForce GTX 1070 core. Keras v.2.2.4 (http://github.com/fchollet/keras), a neural networks API written in Python and developed based on TensorFlow 1.13.1 (https://github.com/tensorflow), was adopted for parallel computing. CNN and DNN models were trained by minimizing cross-entropy loss between final predictions and ground-truth labels. During training, the Adam optimizer in Keras was adopted, and a decay factor *d* was used to control the learning rate at each epoch as shown by the equation

10 $${\mathrm{lr}}_i = {\mathrm{lr}} \times \frac{1}{{1 + d \times i}}$$

Where lr was the initial learning rate and lr_*i*_ was the learning rate at *i*th epoch. Adjustable parameters, such as the dropout ratio, initial learning rate, decay and batch size were simultaneously optimized to improve the performance (Supplementary Data 4). In addition, the CNNs of Inception Net V3^36^ and ChexNet^37^ were obtained directly from Keras. The Adam optimizer was used for model training, with an initial learning rate of 0.0001, a decay of 0.05, a batch size of 64 and epochs of 500. Cohort 2 was adopted as a validation dataset.

### Statistical analysis

For each of the 125 types of numerical CF, the normality of the data distribution was evaluated by the Shapiro–Wilk test, a commonly used normality test, using the stats.shapiro() function in Python 3.7. A threshold of *P* < 0.05 was set for a CF with data not following the normal distribution (Supplementary Data 1). For the 11 CFs with numerical data following the normal distribution, a two-sided unpaired *t*-test was performed using the stats.ttest_ind() function in Python 3.7 (Supplementary Data 3). For the remaining 114 types of numerical CFs with data not following the normal distribution, the two-sided Mann–Whitney *U* test, the nonparametric equivalent to the unpaired *t*-test, was performed using the stats.mannwhitneyu() function in Python 3.7 (Supplementary Data 3). Mean value and s.d. were calculated, and *P* < 10^-4^ was considered as statistically significant. In statistics, mean and s.d. are measures of location and spread, respectively. When the data is sparse with extreme values, mean might not reflect the central location of data points, and s.d. may be high. Thus, mean and s.d. values calculated in this study could be regarded only as a reference. For multiple-hypothesis-testing correction, the adjusted *P*-value (<10^−3^) was calculated using the Benjamini–Hochberg method (Supplementary Data 3). For statistical comparisons of different types of patients with or without Udis, the two-sided chi-squared test was performed using the 2 × 2 table. *χ*^2^ was calculated and the *P*-value (<0.05) was computed using the CHIDIST(*χ*^2^, degree_freedom) function in Excel. The degree_freedom was equal to 1 for each 2 × 2 table (Supplementary Data 3).

### Reporting summary

Further information on research design is available in the Nature Research Reporting Summary linked to this article.

## Supplementary information

Supplementary Information Supplementary figures and descriptions of the dataset.

Reporting Summary

Peer review information

Supplementary Dataset 1 Details about the 9 classes and 130 types of clinical features and of the diseases and morbidity outcomes of the patients, statistical comparisons of clinical features in different types of patient, and hyperparameters of the neural networks.

## Data Availability

The data supporting the results in this study are available within the paper and its Supplementary Information. All source datasets, including chest CT images in both DICOM and JPEG formats, CFs and laboratory confirmations, are archived and maintained at http://ictcf.biocuckoo.cn. The 19,685 manually labelled CT slices in JPEG format, including 5,705 NiCT, 4,001 pCT and 9,979 nCT images, are downloadable from http://ictcf.biocuckoo.cn/HUST-19.php.

## Code availability

All source codes and computational models of HUST-19, Inception Net V3^36^ and ChexNet^37^ are available at http://ictcf.biocuckoo.cn/HUST-19.php.
