# Supplementary material for: Open resource of clinical data from patients with pneumonia for the prediction of COVID-19 outcomes via deep learning
Source: Nat Biomed Eng. 2020 Nov 18;4(12):1197–207. doi: 10.1038/s41551-020-00633-5 (PMC7723858; doi:10.1038/s41551-020-00633-5)
Supplement: Supplementary file 1 — Supplementary figures and descriptions of the dataset. [file 41551_2020_633_MOESM1_ESM.pdf]

nature research

https://doi.org/10.1038/s41551-020-00633-5

Supplementary information

Open resource of clinical data from patients with pneumonia for the prediction of COVID-19 outcomes via deep learning

In the format provided by the authors and unedited

Table of contents

Supplementary Fig. 1 | The most significantly increased and decreased CFs 2
Supplementary Fig. 2 | The resource page of iCTCF database 3
Supplementary Fig. 3 | The accuracy for predicting morbidity or mortality outcomes exclusively using CT or CF data 4
Supplementary Fig. 4 | The accuracy of Inception Net V3 and ChexNet for predicting morbidity or mortality outcomes using our CT data 5
Supplementary Data 1 6
Supplementary Data 2 6
Supplementary Data 3 6
Supplementary Data 4 6

Supplementary figures

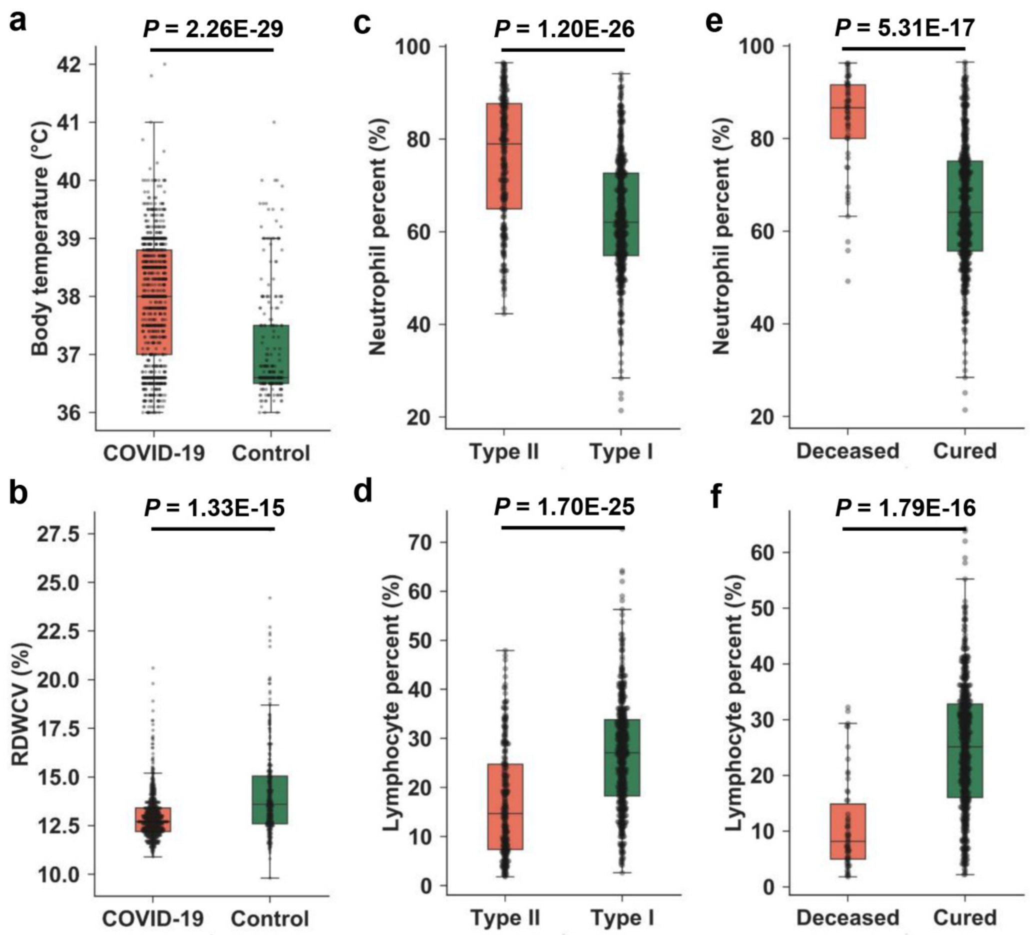

Supplementary Fig. 1 | The most significantly increased and decreased CFs. a,b, The most significantly increased (a) (Body temperature, *P* = 2.26E-29) and decreased (b) (RDWCV, *P* = 1.33E-15) CFs in COVID-19 patients (Type I & II, *n* = 894) against control cases (*n* = 328). **c,d,** The most significantly increased (c) (Neutrophil percent, *P* = 1.20E-26) and decreased (d) (Lymphocyte percent, *P* = 1.70E-25) CFs in Type II patients (*n* = 274) against Type I cases (*n* = 620). **e,f,** The most significantly increased (e) (Neutrophil percent, *P* = 5.31E-17) and decreased (f) (Lymphocyte percent, *P* = 1.79E-16) CFs in deceased cases (*n* = 57) against cured cases (*n* = 662). Box plots denote median and 25th to 75th percentiles (boxes) and 10th to 90th percentiles (whiskers). The data of these CFs did not follow the normal distribution (Supplementary Data 1), and *p*-values were calculated by the two-sided Mann-Whitney U test.

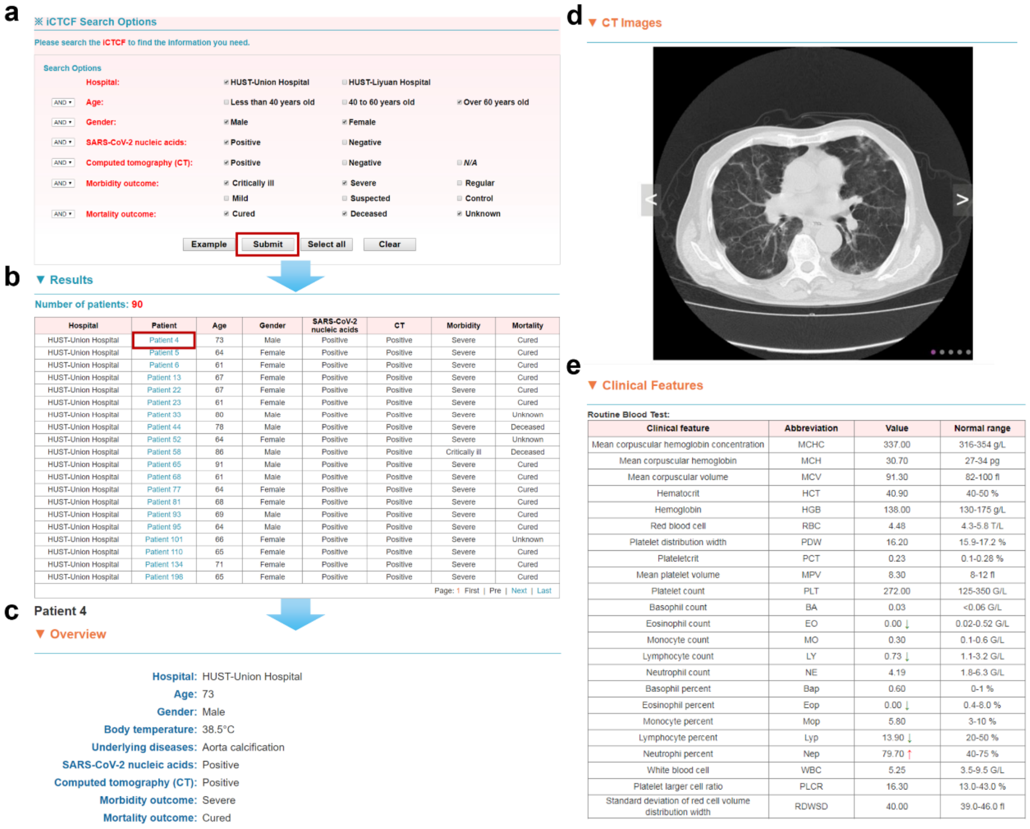

Supplementary Fig. 2 | The resource page of iCTCF database. a, Multiple selection options were provided for searching the resource in a customizable manner. **b,** After clicking “Submit”, the results returned in a tabular list. **c,** A general summary of a given patient in the overview section. **d,** One of five representative chest CT slices in JPEG format was displayed for each patient. The other slices could be seen by clicking horizontal scrolling buttons. **e,** The numerical CFs of a given patient were shown in a tabular list.

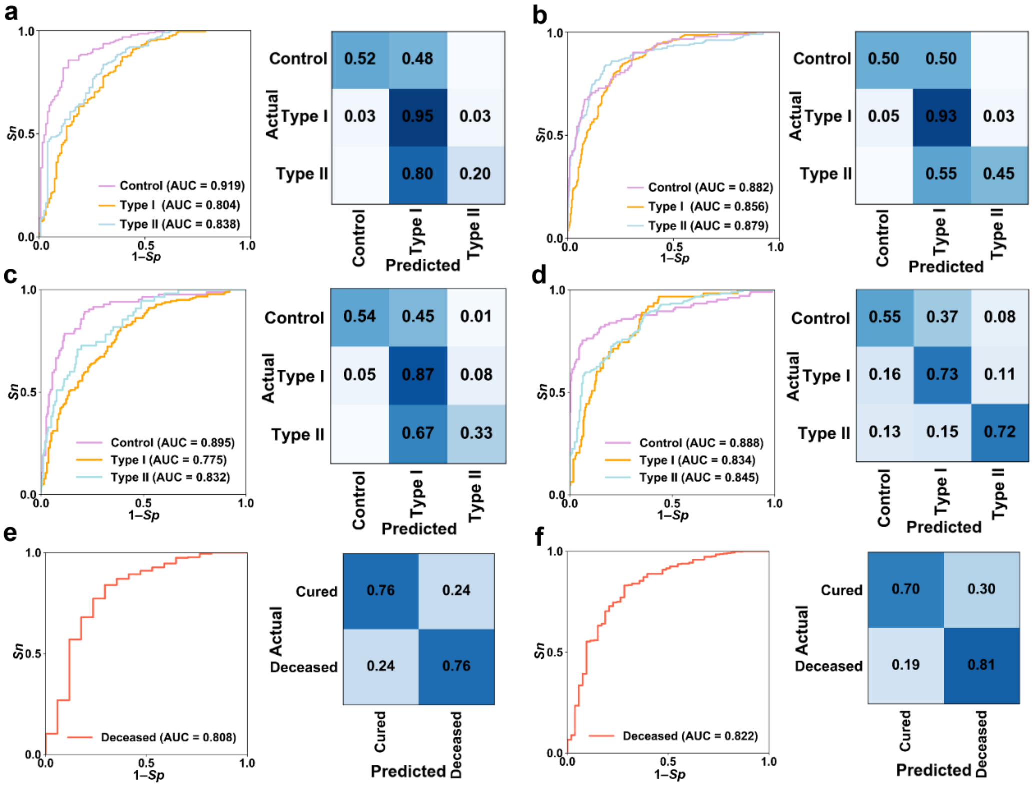

Supplementary Fig. 3 | The accuracy for predicting morbidity or mortality outcomes exclusively using CT or CF data. Both receiver operating characteristic (ROC) curves and confusion matrices were illustrated. **a,** CT-based prediction of morbidity outcomes using the Cohort 1 (207 controls, 384 Type I patients, and 149 Type II patients having CT data). **b,** CF-based prediction of morbidity outcomes using the Cohort 1 (222 controls, 438 Type I patients, and 211 Type II patients having CF data). **c,** CT-based prediction of morbidity outcomes using the Cohort 2 (106 controls, 180 Type I patients, and 56 Type II patients having CT data). **d,** CF-based prediction of morbidity outcomes using the Cohort 2 (106 controls, 182 Type I patients, and 63 Type II patients having CF data). **e,** CT-based prediction of mortality outcomes using the merged Cohort (593 cured and 19 deceased cases having CT data). **f** CF-based prediction of mortality outcomes using the merged Cohort (662 cured and 57 deceased cases having CF data).

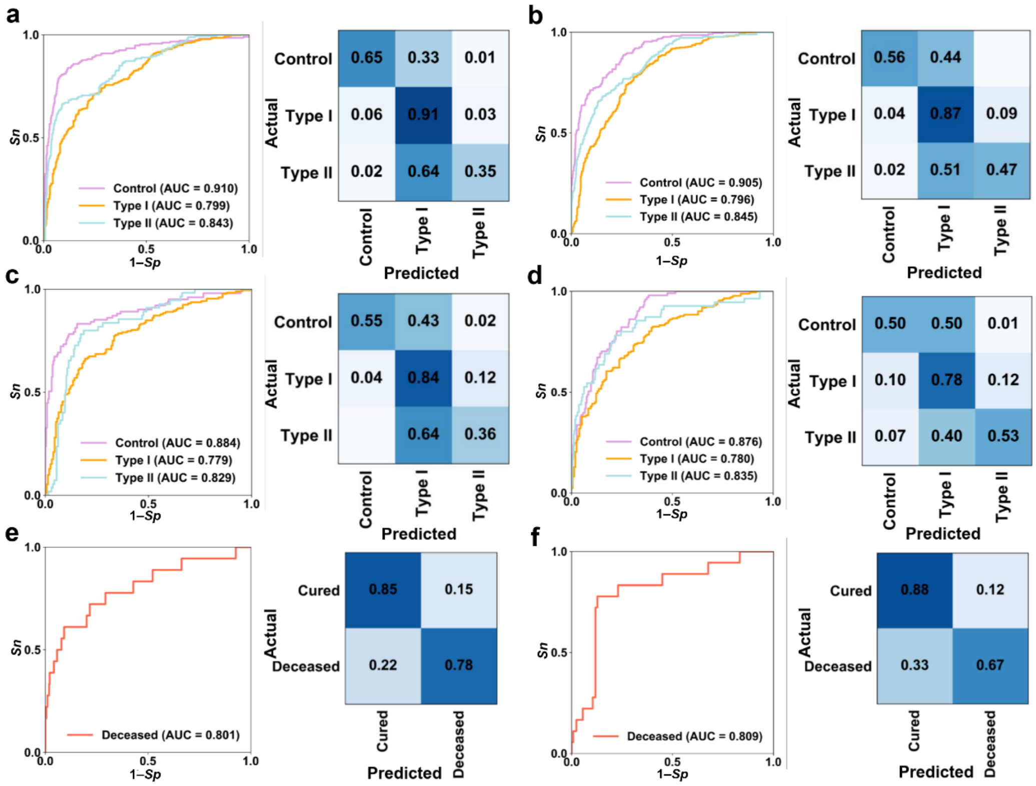

Supplementary Fig. 4 | The accuracy of Inception Net V3[36] and ChexNet[37] for predicting morbidity or mortality outcomes using our CT data. Both ROC curves and confusion matrices were illustrated. **a,b,** The prediction of morbidity outcomes using the Cohort 1 based on Inception Net V3 (a) and ChexNet (b) (207 controls, 384 Type I patients, and 149 Type II patients having CT data). **c,d,** The prediction of morbidity outcomes using the Cohort 2 based on Inception Net V3 (c) and ChexNet (d) (106 controls, 180 Type I patients, and 56 Type II patients having CT data). **e,f,** The prediction of mortality outcomes using the merged Cohort based on Inception Net V3 (e) and ChexNet (f) (593 cured and 19 deceased cases having CT data).

Description of the Supplementary Dataset

Supplementary Data 1

The details on 9 classes and 130 types of CFs, including CF names, abbreviations, and normal range values in HUST-UH and HUST-LH. *a*. Before statistical analysis, the normality of data distribution was evaluated by the Shapiro-Wilk test for each CF. *b*. *p*-value > 0.05 denoted data following normal distribution, and there were 11 CFs following the normal distribution; *c*. *N/A*, not available; *d*. F/M, female or male.

Supplementary Data 2

The information regarding underlying diseases and morbidity outcomes of patients from HUST-UH or HUST-LH for the two cohorts.

Supplementary Data 3

The statistical comparisons of numerical CFs in different types of patients. In the sheet "Positive *vs.* Control", statistically higher and lower (Marked in grey) CFs were shown in cases with COVID-19 pneumonia against control patients. In the sheet "Type II *vs.* Type I", statistically higher and lower (Marked in grey) CFs were shown in Type II patients against Type I patients. In the sheet "Deceased *vs.* Cured", statistically higher and lower (Marked in grey) CFs were shown in deceased cases against cured patients. For the above three sheets, fold changes were present, while the two-sided unpaired t-test and Mann-Whitney U test were conducted for CF data following normal distribution or not, respectively (p-value < 10^{-4}). Adjusted *p*-values were calculated using the Benjamini-Hochberg method (< 10^{-3} in this study). In the sheet "Underlying diseases", the proportions of patients with and without underlying diseases (Udis) were compared (Two-sided chi-squared test) between COVID-19 patients (Type I & II) and controls, between Type II and Type I patients, and between deceased and cured cases.

Supplementary Data 4

All pre-configured parameters in CNN and DNN frameworks. *a*. Input, size of an input image (height * width * channel); *b*. Convolutional, number and size of convolutional kernels (number * height * width); *c*. Pooling, size of a pooling layer (height * width); *d*. Dense, number of nodes in the dense layer; *e*. Dropout, the dropout ratio; *f*. Output, number of nodes in the output layer; *g*. Decay, learning rate decay factor; *h*. Epochs, number of iterations.
