# Supplementary material for: Open resource of clinical data from patients with pneumonia for the prediction of COVID-19 outcomes via deep learning
Source: Nat Biomed Eng. 2020 Nov 18;4(12):1197–207. doi: 10.1038/s41551-020-00633-5 (PMC7723858; doi:10.1038/s41551-020-00633-5)
Supplement: Supplementary file 2 — Reporting Summary [file 41551_2020_633_MOESM2_ESM.pdf]

nature research

Corresponding author(s): Zheng Wang, Yu Xue, Lin Wang, Heshui Shi, Yulan Zeng

Last updated by author(s): Sep 16, 2020

Reporting Summary

Nature Research wishes to improve the reproducibility of the work that we publish. This form provides structure for consistency and transparency in reporting. For further information on Nature Research policies, see Authors & Referees and the Editorial Policy Checklist.

Statistics

For all statistical analyses, confirm that the following items are present in the figure legend, table legend, main text, or Methods section.

n/a	Confirmed	
☐	☒	The exact sample size (n) for each experimental group/condition, given as a discrete number and unit of measurement
☐	☒	A statement on whether measurements were taken from distinct samples or whether the same sample was measured repeatedly
☐	☒	The statistical test(s) used AND whether they are one- or two-sided<br>*Only common tests should be described solely by name; describe more complex techniques in the Methods section.*
☒	☐	A description of all covariates tested
☐	☒	A description of any assumptions or corrections, such as tests of normality and adjustment for multiple comparisons
☐	☒	A full description of the statistical parameters including central tendency (e.g. means) or other basic estimates (e.g. regression coefficient) AND variation (e.g. standard deviation) or associated estimates of uncertainty (e.g. confidence intervals)
☐	☒	For null hypothesis testing, the test statistic (e.g. F, t, r) with confidence intervals, effect sizes, degrees of freedom and P value noted<br>*Give P values as exact values whenever suitable.*
☒	☐	For Bayesian analysis, information on the choice of priors and Markov chain Monte Carlo settings
☒	☐	For hierarchical and complex designs, identification of the appropriate level for tests and full reporting of outcomes
☒	☐	Estimates of effect sizes (e.g. Cohen's d, Pearson's r), indicating how they were calculated

Our web collection on statistics for biologists contains articles on many of the points above.

Software and code

Policy information about availability of computer code

Data collection	No software was used.
Data analysis	The iCTCF database was developed via PHP 7.0.33 (https://www.php.net/), HTML: XHTML 1.0 Transitional (https://www.w3.org/TR/xhtml1/) and JavaScript (https://www.javascript.com/). The preprocessing of chest computed tomography (CT) images was performed by OpenCV 3.4.2 (https://opencv.org/) and Scikit-image 0.15.0 (https://scikit-image.org/). The Keras 2.2.4 (http://github.com/fchollet/keras) based on Tensorflow 1.13.1 (https://github.com/tensorflow) backend was adopted for the testing and training of the convolutional neural network (CNN) and deep neural network (DNN) models. Scikit-learn 0.21.2 (https://scikit-learn.org/stable/) was adopted for the training and testing of penalized logistic regression (PLR) models, and for t-distributed stochastic neighbor embedding (t-SNE) analysis.

For manuscripts utilizing custom algorithms or software that are central to the research but not yet described in published literature, software must be made available to editors/reviewers. We strongly encourage code deposition in a community repository (e.g. GitHub). See the Nature Research guidelines for submitting code & software for further information.

Data

Policy information about availability of data

All manuscripts must include a data availability statement. This statement should provide the following information, where applicable:

- Accession codes, unique identifiers, or web links for publicly available datasets
- A list of figures that have associated raw data
- A description of any restrictions on data availability

The data supporting the results in this study are available within the paper and its Supplementary Information. All source datasets, including chest CT images in both DICOM and JPEG formats, CFs and laboratory confirmations, are archived and maintained at http://ictcf.biocuckoo.cn. Manually labelled 19,685 CT slices in JPEG format, including 5705 NiCT, 4001 pCT and 9979 nCT images, are downloadable from http://ictcf.biocuckoo.cn/HUST-19.php.

Field-specific reporting

Please select the one below that is the best fit for your research. If you are not sure, read the appropriate sections before making your selection.

☒ Life sciences ☐ Behavioural & social sciences ☐ Ecological, evolutionary & environmental sciences

For a reference copy of the document with all sections, see nature.com/documents/nr-reporting-summary-flat.pdf

Life sciences study design

All studies must disclose on these points even when the disclosure is negative.

Sample size	The study included 1,521 patients: 1,126 patients from HUST-UH and 395 from HUST-LH. The data were from (i) patients receiving RT-PCR nucleic acid testing and hospitalized between Jan. 25, 2020, and Feb. 29, 2020, at HUST-UH and HUST-LH; (ii) patients admitted to HUST-UH between Aug. 20, 2019, and Nov. 30, 2019, and diagnosed with community-acquired pneumonia; (iii) healthy cases from routine physical check-ups. All the cases in the dataset were independent and non-repeating. We used all samples that we could collect from the two hospitals. We deemed the sample size sufficient, as we achieved promising accuracies on both the training and validation cohorts.
Data exclusions	No data were excluded.
Replication	10-fold cross-validations were performed to evaluate the prediction accuracy of HUST-19. We also used an independent validation dataset. The source code of HUST-19 is provided at http://ictcf.biocuckoo.cn/HUST-19.php. All primary data were checked by at least three researchers, and all results are reproducible.
Randomization	Randomization was not applicable because the study was retrospective.
Blinding	Blinding was not applicable, because the study was retrospective.

Reporting for specific materials, systems and methods

We require information from authors about some types of materials, experimental systems and methods used in many studies. Here, indicate whether each material, system or method listed is relevant to your study. If you are not sure if a list item applies to your research, read the appropriate section before selecting a response.

Materials & experimental systems

n/a	Involved in the study	
☒	☐	Antibodies
☒	☐	Eukaryotic cell lines
☒	☐	Palaeontology
☒	☐	Animals and other organisms
☐	☒	Human research participants
☒	☐	Clinical data

Methods

n/a	Involved in the study	
☒	☐	ChIP-seq
☒	☐	Flow cytometry
☒	☐	MRI-based neuroimaging

Human research participants

Policy information about studies involving human research participants

Population characteristics	We enrolled 1,521 patients: 1,126 patients from HUST-UH and 395 from HUST-LH. The dataset consisted of patients aged 1–97 (mean ± standard deviation: 54.85 years ± 17.34 years), 756 males and 765 females. All patients had CF data, and 1,342 patients had both CT and CF data. On the basis of the China Guidance for COVID-19 (6th edition), the clinical morbidity outcomes of the 1,521 patients were classified as (i) 894 COVID-19-confirmed patients with pneumonia severity classified as mild (24 cases, 2.7%), regular (596 cases, 66.7%), severe (202 cases, 22.6%), and critically ill (72 cases, 8.1%); (ii) 328 COVID-19-negative cases (regarded as controls); and (iii) 299 COVID-19 suspected cases. For the COVID-19-confirmed patients, their mortality outcomes were also counted: 662 cured cases, 57 deceased cases and 175 cases with unknown outcomes (these patients transferred to other hospitals).
Recruitment	No patient recruitment was performed, as the study was retrospective. The CT and CF data of the COVID-19 patients receiving RT-PCR nucleic acid testing and hospitalized between Jan. 25, 2020, and Feb. 29, 2020, were collected at HUST-UH and HUST-LH.
Ethics oversight	The collection, use and retrospective analyses of chest CT images, CFs and SARS-CoV-2 nucleic acid PCR results of the patients were approved by the institutional ethical committees of HUST-UH (IRB ID: [2020] IEC (A001)) and HUST-LH (IRB ID: [2020] IEC (A001)).

Note that full information on the approval of the study protocol must also be provided in the manuscript.
