# Supplementary material for: Open resource of clinical data from patients with pneumonia for the prediction of COVID-19 outcomes via deep learning
Source: Nat Biomed Eng. 2020 Nov 18;4(12):1197–207. doi: 10.1038/s41551-020-00633-5 (PMC7723858; doi:10.1038/s41551-020-00633-5)
Supplement: Supplementary file 3 — Peer review information [file 41551_2020_633_MOESM3_ESM.pdf]

nature biomedical engineering PEER-REVIEW INFORMATION

Open resource of clinical data from patients with pneumonia for the prediction of COVID-19 outcomes via deep learning

Corresponding author: Yu Xue

Editorial note

This document includes relevant written communications between the manuscript's corresponding author and the editor and reviewers of the manuscript during peer review. It includes decision letters relaying any editorial points and peer-review reports, and the authors' replies to these (under 'Rebuttal' headings). The editorial decisions are signed by the manuscript's handling editor, yet the editorial team and ultimately the journal's Chief Editor share responsibility for all decisions.

Any relevant documents attached to the decision letters are referred to as **Appendix #**, and can be found appended to this document. Any information deemed confidential has been redacted or removed. Earlier versions of the manuscript are not published, yet the originally submitted version may be available as a preprint. Because of editorial edits and changes during peer review, the published title of the paper and the title mentioned in below correspondence may differ.

New machine-learning experts also knowledgeable about clinical radiology were recruited to review revised versions of the manuscript.

Two recruited reviewers (Reviewer #1 and Reviewer #5) did not deliver any report.

Correspondence

Tue 31/03/2020
Request for the reporting summary and policy checklist for Article nBME-20-0693

Dear Prof Xue,

Thank you for submitting to *Nature Biomedical Engineering* your Article, "iCTCF: an integrative resource of chest computed tomography images and clinical features of patients with COVID-19 pneumonia". Having assessed the work, we have decided to send the manuscript out to external review. However, before we contact potential reviewers, I should ask you to please fill in our reporting summary and policy checklist. (Please note that the reporting summary will be available to the reviewers, and these forms are dynamic PDF files that can only be properly visualized and filled in by using Acrobat Reader.)

Both documents are aimed at ensuring good reporting standards as well as compliance with policies on research ethics. Should the manuscript be published, the reporting summary will be attached to the PDF version of the paper and will also be available as supplementary information. More information is available on the editorial policies page.

Please note that the code will need to be peer-reviewed, and validated by the reviewers with additional datasets from another country.

Also, please update the submitted manuscript files should you need to modify the manuscript to include any missing information on statistics or methods or to update the plots to conform to the recommended data-presentation policies.

Please note that we will need you to provide, in the manuscript, additional information in regards to the machine learning section. The current version of your study provides the AUC values but in order for us and the reviewers to evaluate the performance of your machine learning classification you should provide the positive and negative predictive values, sensitivity and specificity values, in a confusion matrix. Please note that the characteristics of the test dataset in the current manuscript are unclear, the process you use for t-

distributed stochastic neighbour embedding should be clearly described, as well as the process used for the manual labelling and consensus.

When you have completed both the reporting summary and the checklist, please upload both forms as well as any updated manuscript files. (*The link in this paragraph will allow you to securely send the files to us via Dropbox. You do not need to have a Dropbox account. Please make sure that each filename follows the pattern ####CorrespondingAuthorSurname_FileDescription, where #### are the four last digits of the manuscript tracking number, and note that we will get automatically notified when your files have been uploaded*.)

Best wishes,

Rosy

__
Dr Rosy Favicchio
Senior Editor, *Nature Biomedical Engineering*

Tue 28/04/2020
Decision on Article nBME-20-0693

Dear Prof Xue,

Thank you again for submitting to *Nature Biomedical Engineering* your Article, "iCTCF: an integrative resource of chest computed tomography images and clinical features of patients with COVID-19 pneumonia". The manuscript has been seen by three experts, whose reports you will find at the end of this message. We are still waiting for the fourth reviewer to send a report and we will forward it to you as soon as we have it. You will see that although the reviewers have some good words for the work, they articulate concerns about the degree of support for the claims, and in this regard provide useful suggestions for improvement. We hope that with significant further work you can address the criticisms and convince the reviewers of the merits of the study. In particular, we would expect that a revised version of the manuscript provides:

* improved image data analysis that rectifies the inconsistencies between datasets, as described by Reviewer #3 (please refer to the attached files).

* access to the DICOM files, as well as any other image processing information required to reproduce the methodology, for each patient in your database.

* rationale that explains the choice of jpeg as a file format for the computational analysis.

* an assessment of the performance of the trained algorithm in an independent dataset.

* a correlation between the type of pneumonia recorded with morbidity and mortality outcomes.

When you are ready to resubmit your manuscript, please upload the revised files, a point-by-point rebuttal to the comments from all reviewers, the (revised, if needed) reporting summary, and a cover letter that explains the main improvements included in the revision and responds to any points highlighted in this decision.

Please follow the following recommendations:

* Clearly highlight any amendments to the text and figures to help the reviewers and editors find and understand the changes (yet keep in mind that excessive marking can hinder readability).

* If you and your co-authors disagree with a criticism, provide the arguments to the reviewer (optionally, indicate the relevant points in the cover letter).

* If a criticism or suggestion is not addressed, please indicate so in the rebuttal to the reviewer comments and explain the reason(s).

* Consider including responses to any criticisms raised by more than one reviewer at the beginning of the rebuttal, in a section addressed to all reviewers.

* The rebuttal should include the reviewer comments in point-by-point format (please note that we provide all reviewers will the reports as they appear at the end of this message).

* Provide the rebuttal to the reviewer comments and the cover letter as separate files.

We hope that you will be able to resubmit the manuscript within 25 weeks from the receipt of this message. If this is the case, you will be protected against potential scooping. Otherwise, we will be happy to consider a revised manuscript as long as the significance of the work is not compromised by work published elsewhere or accepted for publication at *Nature Biomedical Engineering*. Because of the COVID-19 pandemic, should you be unable to carry out experimental work in the near future we advise that you reply to this message with a revision plan in the form of a preliminary point-by-point rebuttal to the comments from all reviewers that also includes a response to any points highlighted in this decision. We should then be able to provide you with additional feedback.

We hope that you will find the referee reports helpful when revising the work. Please do not hesitate to contact me should you have any questions.

Best wishes,

Rosy

__
Dr Rosy Favicchio
Senior Editor, *Nature Biomedical Engineering*

____

Reviewer #2 (Report for the authors (Required)):

* A brief summary of the results.
The authors frame the COVID-19 pandemic with the premise that computed tomography (CT) datasets would aid clinical decision imaging for early diagnosis. The authors enrolled 1170 patients, including 649 confirmed, 22 negative, and 299 suspected patients while collecting relevant imaging (CT), clinical features, and SARS CoV-2 test results. The authors aim to predict negative, mild, and severe cases. The authors note various differences in clinical features between negative and COVID cases in addition to segmentation between Type I and Type II cases. They also developed a resource to share CTs and CFs, as well as lab testing – available online. They then developed HUST-19, computational method to integrate and predict Type of patient based on CT/CF/lab data. The strongest result was integration of CT and CFs datasets resulting in AUC values of 0.978, 0.921 and 0.931 in predicting controls, Type I and II patients, respectively.

* Your reasoned opinion on the degree of advance (fundamental, mechanistic, methodological, technological, therapeutic, translational and/or clinical) of the work with respect to the state of the art. If the results or conclusions are not original, please provide relevant references.

The authors should firstly be commended for their efforts in contributing an open-access repository of imaging and data during the pandemic – this will aid others in research. This paper, while interesting, can be categorized as of minimal clinical advancement in the field of radiology and medicine in its current form. It should be noted that imaging is not recommended by any leading radiological society to rule out COVID-19 pneumonia. Please refer to the American College of Radiology (https://www.acr.org/Advocacy-and-Economics/ACR-Position-Statements/Recommendations-for-Chest-Radiography-and-CT-for-Suspected-COVID19-Infection). Ultimately, clinicians are not treating the imaging, they are treating the patient. This is not to say that the results are not impressive, but to note they are unlikely to contribute a fundamental clinical impact. Should there be additional impact of the algorithm, such as morbidity and mortality, this would be improved.

The data on clinical feature patients (192 out of 1170) is limited. Moreover, the authors segment cases into negative, Type I, and Type 2, however do not reference which scale they are using or how their methods of review and type characterization (blinded radiologists?). Without a validated methodology to categorize severity the premise of this paper claiming clinical relevance to the clinician is minimized. The paper notes “researchers” and “clinicians” (could clinicians mean radiologists?) examined cases of HUST-19, however as the standard this should be done only by radiologists, who are the imaging experts. Moreover, what are the radiographic criteria that constitute “mild” and “severe”? This paper would be improved by correlating Type of pneumonia with morbidity and mortality outcomes.

* Your reasoned opinion on the broad implications of the findings.

As above, because of the fundamental nature of COVID pneumonia, this paper is likely of minimal clinical relevance.

As numbered lists:

* Any major technical criticisms or questions.
1. Lack of a proper gold standard by a radiologist (this paper notes “researchers” and “clinicians” labelled the CTs -unless these were radiologists this classification should be redone)
2. Limited dataset of clinical features
3. Lack of reference or validated methodology to categorize Type I vs Type II.
4. Lack fo radiographic features categorizing Type I vs Type II

* Any minor technical criticisms or questions.
1. “radiographic dataset” should be referred to as “CT dataset” – most will interpret radiographic to mean chest xray, which is of prevalent use in the COVID pandemic.
2. “Linear shadows” is not a radiographic term applicable to CT scans, please remove or rephrase.

* Any missing or unclear details about statistics, protocols or materials (please check the reporting checklist provided with the manuscript files).
I felt this was well researched, no missing details

* Any missing citations to relevant literature (please keep in mind that the suggested maximum number of references is 50).
N/A

* Any optional suggestions for improvement.
1. Clarify who reviewed the CT scans and what their expertise is
2. Validate your Type reporting method or reference the validated methodology
3. Consider extending implications to morbidity and mortality outcomes, or length of stay, to make this more clinically relevant.

* Any stylistic issues or recommendations.
-Grammatical and spelling errors throughout
-Please correct the tense, varies between past and present
-iCTCF needs to be defined on first use in the abstract

Reviewer #3 (Report for the authors (Required)):

See attached file.
Appendix 1

Reviewer #4 (Report for the authors (Required)):

Thank you very much for submitting your work. The authors integrated the heterogeneous CT and CF datasets, and developed a novel framework of Hybrid-learning for UnbiaSed predicTion of COVID-19 patients (HUST-19). The team have put a large database together and freely sharing it with the research community under the CC-BY-NC license. This is of great meaning considering the emerging situation. However, there are few questions that need to be resolved：
1. For the study, the authors defined mild and regular forms as Type I, and severe and critically ill forms as Type II. Are there differences between them? Is the clinical treatment consistent? Whether they are combined analysis is reasonable?

2. Almost all statistical test methods are t-test, whether the data distribution meets the statistical requirements？

3. I'm not sure if 10-fold cross-validations are enough to demonstrate the credibility, is the suspected patients used as a validation dataset, whether an external validation dataset is needed?

4. “Here, we used HUST-19 with the sensitive threshold to predicted 21,207 and 71 patients of 299 suspected cases to be COVID-19 negative cases, Type I cases, and Type II cases, respectively.” should be “Here, we used HUST-19 with the sensitive threshold to predicted 21, 207 and 71 patients of 299 suspected cases to be COVID-19 negative cases, Type I cases, and Type II cases, respectively.” Missing a comma.

5. Make sure references are up-to-date since this is a rapidly evolving topic. More information may be available at present.

Thu 06/08/2020
Decision on Article NBME-20-0693A

Dear Professor Xue,

Thank you for your revised manuscript, "iCTCF: an integrative resource of chest computed tomography images and clinical features of patients with COVID-19 pneumonia", which has been seen by two more experts in machine learning. In their reports, which you will find at the end of this message, you will see that the reviewers acknowledge the improvements to the work and raise a few additional technical criticisms that we hope you will be able to address. Please note that despite our chasing efforts one referee is still to provide feedback, if we do receive comments we will forward them to you. In particular, we would expect that the next version of the manuscript provides

* the remaining minor points raised by the referees, in particular improve the technical accuracy of the language used in the text.

* a detailed methodological section that clarifies the rationale used for the design of the CNN architecture and the rationale for dividing the patients into Type I and Type II.

* an improved description of the statistical section of the manuscript.

As before, when you are ready to resubmit your manuscript, please upload the revised files, a point-by-point rebuttal to the comments from all reviewers, the (revised, if needed) reporting summary, and a cover letter that explains the main improvements included in the revision and responds to any points highlighted in this decision.

As a reminder, please follow the following recommendations:

* Clearly highlight any amendments to the text and figures to help the reviewers and editors find and understand the changes (yet keep in mind that excessive marking can hinder readability).

* If you and your co-authors disagree with a criticism, provide the arguments to the reviewer (optionally, indicate the relevant points in the cover letter).

* If a criticism or suggestion is not addressed, please indicate so in the rebuttal to the reviewer comments and explain the reason(s).

* Consider including responses to any criticisms raised by more than one reviewer at the beginning of the rebuttal, in a section addressed to all reviewers.

* The rebuttal should include the reviewer comments in point-by-point format (please note that we provide all reviewers will the reports as they appear at the end of this message).

* Provide the rebuttal to the reviewer comments and the cover letter as separate files.

We hope that you will be able to resubmit the manuscript within 12 weeks from the receipt of this message. If this is the case, you will be protected against potential scooping. Otherwise, we will be happy to consider a revised manuscript as long as the significance of the work is not compromised by work published elsewhere or accepted for publication at *Nature Biomedical Engineering*. Because of the COVID-19 pandemic, should you be unable to carry out experimental work in the near future we advise that you reply to this message with a revision plan in the form of a preliminary point-by-point rebuttal to the comments from all reviewers that also includes a response to any points highlighted in this decision. We should then be able to provide you with additional feedback.

We look forward to receive a further revised version of the work. Please do not hesitate to contact me should you have any questions.

Best wishes,

Rosy

__
Dr Rosy Favicchio

Senior Editor, *Nature Biomedical Engineering*

Reviewer #2 (Report for the authors (Required)):

The authors frame the COVID-19 pandemic with the premise that computed tomography (CT) datasets would aid clinical decision imaging for early diagnosis. The authors enrolled 1170 patients, including 649 confirmed, 22 negative, and 299 suspected patients while collecting relevant imaging (CT), clinical features, and SARS CoV-2 test results. The authors aim to predict negative, mild, and severe cases. The authors note various differences in clinical features between negative and COVID cases in addition to segmentation between Type I and Type II cases. They also developed a resource to share CTs and CFs, as well as lab testing – available online. They then developed HUST-19, computational method to integrate and predict Type of patient based on CT/CF/lab data. The strongest result was integration of CT and CFs datasets resulting in AUC values of 0.978, 0.921 and 0.931 in predicting controls, Type I and II patients, respectively.

The authors have addressed most of the points raised with this revision. Unfortunately, the provided Severity rating scale link is no in English. My main comment was based around lack of description on who assessed the CT scans. Based on the updated revision, the authors have noted radiologists indeed reviewed the cases. I think given the guidelines differ vs those in the USA, the applicability may be somewhat more limited depending on the region of the reader.

Would suggest the word "shadows" is removed from the revised methods as this is not in the CT radiology lexicon.

Overall, the authors should be commended for gracefully responding to comments and making the suggested changes and clarifications.

Reviewer #3 (Report for the authors (Required)):

The contributions of this paper are two-fold: a large dataset of CT scans and clinical features for COVID-19 suspects and cases is made freely available for research and software to classify subjects based on CT and clinical features has been developed and is described and also shared. In The revision of the paper is much improved with respect to the dataset. The authors have now added the DICOM data and this has removed most of my concerns with the initial version. Moreover, the dataset has been extended by adding Cohort 2.

Concerns about the data

Please specify for the controls in Cohort 1 which cases are normal and which are community acquired pneumonia.

Cohort 2 is named an independent dataset but this is not correct as the data in Cohort 2 is from the same hospitals as the data from Cohort 1. Just call it test set or validation set. List this as a limitation.

Comments about the classification experiments

The abstract says the combined analysis of CT and CF is superior to either one alone, but the results that show this are only in a supplement. I would suggest adding a table in the main paper giving the characteristics of the dataset, and another table with all the experiments and results.

While I see the use of attempting to predict RT-PCR result from CT and lab values, in situations where it is time-consuming to obtain these test results, and in situations where an initial test is negative but clinical suspicion of COVID-19 is high, I think there is no use for a system that predicts mild/regular versus either normal or severe/critical disease.

Reviewer #4 (Report for the authors (Required)):

No additional comments. I thank the authors for being responsive in addressing the reviewer's comments and suggestions. Congratulations!

Reviewer #6 (Report for the authors (Required)):

very interesting study.

provided a comprehensive resource named integrative computed tomography (CT) images and clinical features for COVID-19 (iCTCF) to archive chest CT images, 130 types of CFs, and laboratory-confirmed SARS-CoV-2 clinical status from 1521 patients with or without COVID-19 pneumonia.

Authors integrated the heterogeneous CT and CF datasets: interesting.

Developed a nice piece of engineering work on Hybrid-learning for UnbiaSed predicTion of COVID-19 patients (HUST-19) to predict morbidity and mortality outcomes. Unlike authors claim with the term "novel", I would suggest a nice piece of engineering works, since most of the methods were taken from the existing sources and integrated.

Their integration of CT and CF datasets achieves interesting performance.

A very few, but major comments:
+ How authors did come up with 13-layered CNN?
+ Also, can we have clear explanation about how did those hyper parameters managed for this interesting task?
+ How about custom CNN, inceptionNet (https://link.springer.com/article/10.1007/s13246-020-00888-x), or ChexNet?
+ Statistical analysis needs need to be extended. Take-home message must be clear from this section.

# Clarity about the meaning of mean and standard deviation by taking sparsity (of the data) into account.
How significant the test results are?
Can we take a look at statistical tests?

Dataset collection is fairly large and happy to see that for computational scientists. Can they be available for research purpose? Meaning, reproducible materials will be great.

Overall, interesting paper, and would like to review revised version, if necessary.

Thu 10/09/2020
Decision on Article NBME-20-0693B

Dear Professor Xue,

Thank you for your revised manuscript, "iCTCF: an integrative resource of chest computed tomography images and clinical features of patients with COVID-19 pneumonia". Having consulted with Reviewers #2, #3 and #6, I am pleased to say that we shall be happy to publish the manuscript in *Nature Biomedical Engineering*, provided that the points specified in the attached instructions file are addressed.

When you are ready to submit the final version of your manuscript, please upload the files specified in the instructions file.

For primary research originally submitted after December 1, 2019, we encourage authors to take up transparent peer review. If you are eligible and opt in to transparent peer review, we will publish, as a single supplementary file, all the reviewer comments for all the versions of the manuscript, your rebuttal letters, and the editorial decision letters. **If you opt in to transparent peer review, in the attached file please tick the box 'I wish to participate in transparent peer review'; if you prefer not to, please tick 'I do NOT wish to participate in transparent peer review'.** In the interest of confidentiality, we allow redactions to the rebuttal letters and to the reviewer comments. If you are concerned about the release of confidential data, please indicate what specific information you would like to have removed; we cannot incorporate redactions for any other reasons. If any reviewers have signed their comments to authors, or if any reviewers explicitly
agree to release their name, we will include the names in the peer-review supplementary file. More information on transparent peer review is available.

Please do not hesitate to contact me should you have any questions.

Best wishes,

Rosy

__
Dr Rosy Favicchio
Senior Editor, *Nature Biomedical Engineering*

Reviewer #2 (Report for the authors (Required)):

The authors have satisfactorily addressed my initial comments. I commend the authors on their response.

Reviewer #3 (Report for the authors (Required)):

The authors have adequately addressed all my concerns. In my opinion, the comments from the other reviewers were also well addressed.

Reviewer #6 (Report for the authors (Required)):

No more issues; well-revised paper. The paper can be taken for publication.

Appendix 1

I reviewed this paper and looked primarily at the CT data and the software. I am an expert in thoracic CT image analysis and I do not know much about blood markers.

The HUST-19 database is an excellent resource and the authors should be lauded for building such a resource under such difficult circumstances during a healthcare crisis in a region in lockdown. Sharing HUST-19 with a CC-BY-NC license makes a valuable contribution. It is important to provide the data to the research community as soon as possible. I do not know of any other dataset similar in size that is publicly available. Many groups are looking for data and developing software with small low-quality datasets.

The main limitation of HUST-19 is the fact that the CT data is not provided in DICOM format. Providing jpg images of individual slices severely limits the possibilities for data processing. Reasons for this is that the pixel size is now not available, and the slice thickness is not provided (see also below). The 3D geometry of the scan can therefore only be guessed. I downloaded the 980 zip files and made a simple guess that each scan has a height of 500 pixels, relative to a size of 512x512 pixels per axial slice (note that many scans do not have images that are 512x512, many are larger, and patients 110, 220, 230, 227, 208 contain a jpg of the scoutview of the study, these scoutview images should be removed). I resized all jpgs to 512x512 and constructed 3D volumes and made collages of axial, coronal and sagittal slices to analyze the scans.

In converting to jpg also the Hounsfield Units (a 16 bit scale) are lost (jpg is 8 bit). It is clear from visual inspection that the window-level at which the CT scan was displayed on the jpgs was not the same for every scan. Here are two examples, case 697 and 698:

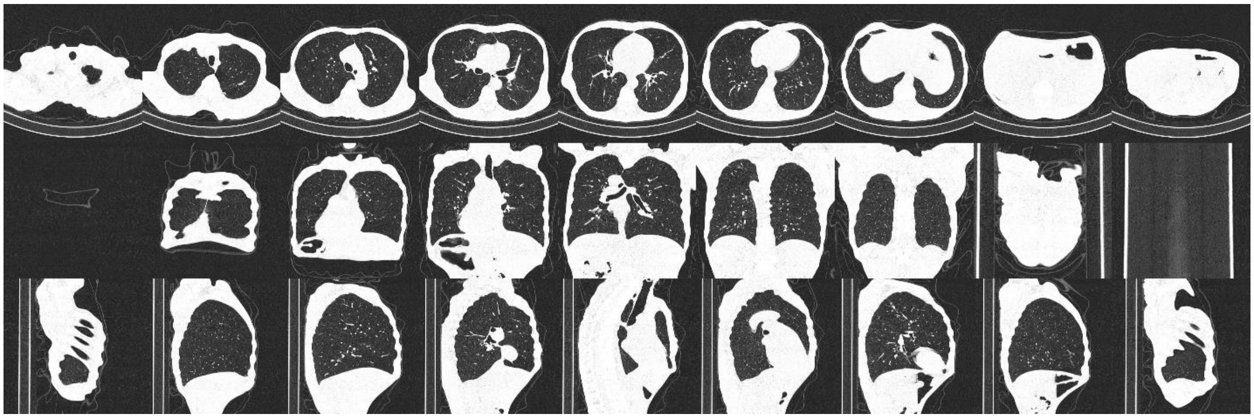

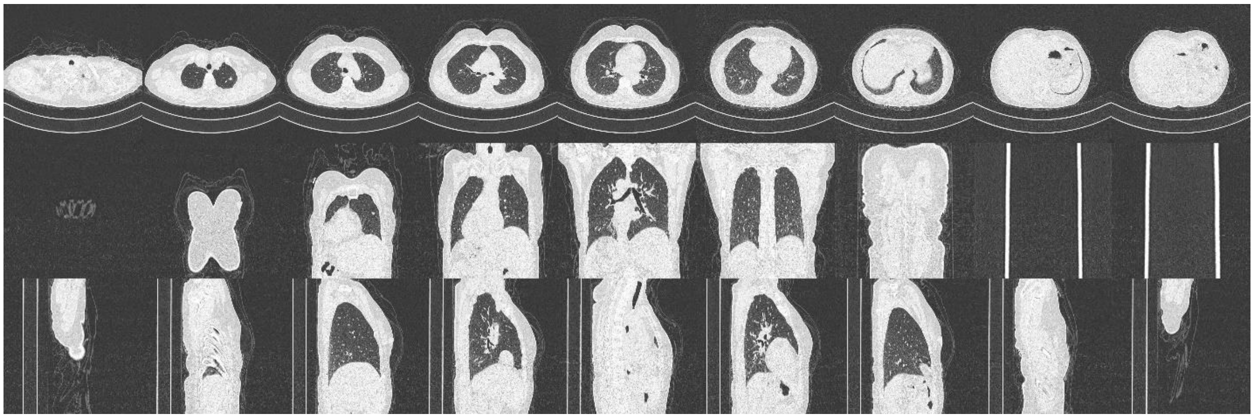

In case 697 much of the mediastinum and abdomen is 255, pure white, while 698 uses a better window level probably close to a recommended setting of 1500/-500 W/L that is often used when inspecting the lung parenchyma on chest CT. With this setting soft tissues (muscle, fat) and bone can still be somewhat differentiated. As the window level setting is not known, it is not possible to accurately estimate Hounsfield Units from the data and many image processing algorithms rely on the fact that CT uses a calibrated density scale and will not work well on this data. Likewise, the deep learning algorithms trained on this data set will only work well on other CT data that is scaled, using this unknown procedure, to jpg images.

Inspecting the data, I found 135/980 cases with a visually clearly deviating window level (too bright mostly, there were also some cases with an abdominal window).

There are also 68 scans with missing slices. This includes 15 studies with only 2 jpgs, these should be removed and three studies with 10, 12, and 12 slices. An example of a case with missing slices is 73:

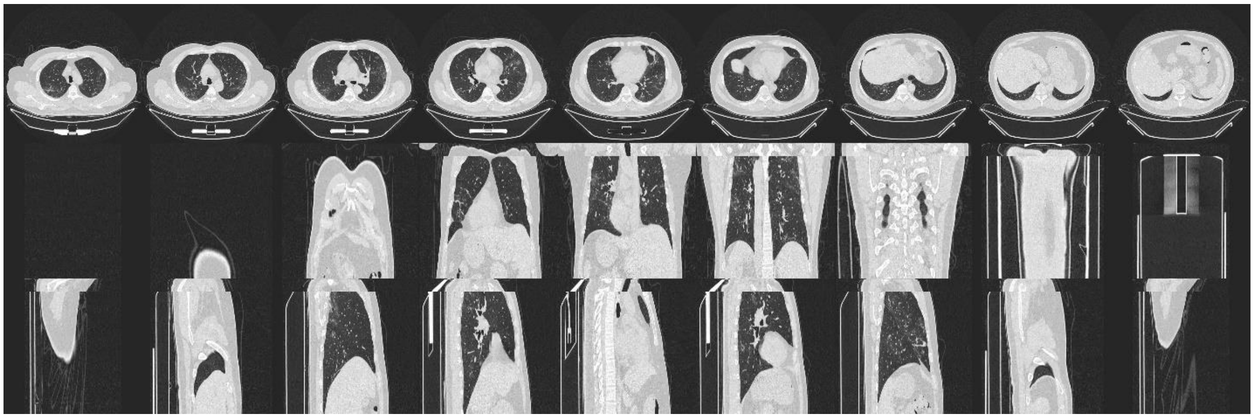

Note the discontinuity in the coronal and sagittal views.

6 studies are upside down. This is easily fixed of course.

I recommend to retrieve the DICOM data and share these. This should solve these issues. In its current form, the resource may also be useful, but much less so.

There are also limitations that would not be resolved even if DICOM would be available. 41 studies do not fully contain the lungs (field of view for the scan was not properly set. An example is patient 1160:

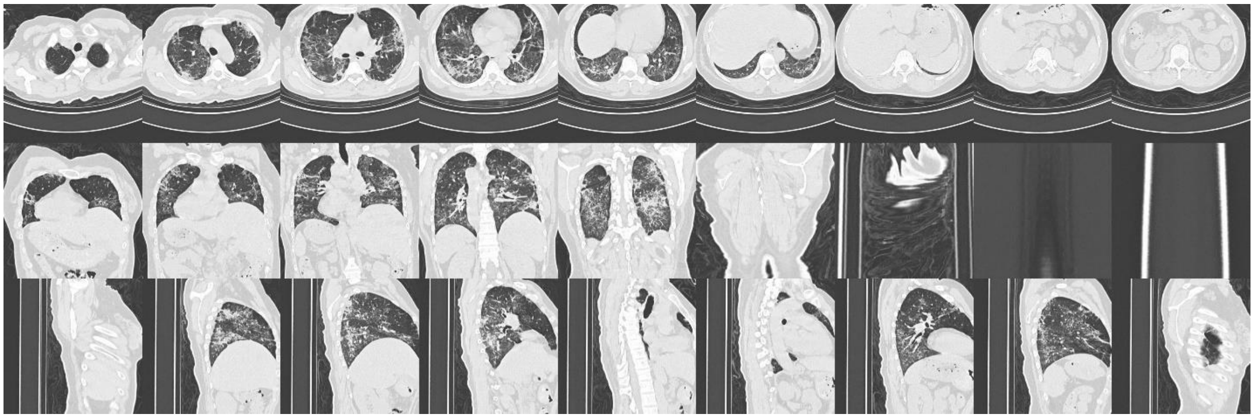

A severe example is patient 874:

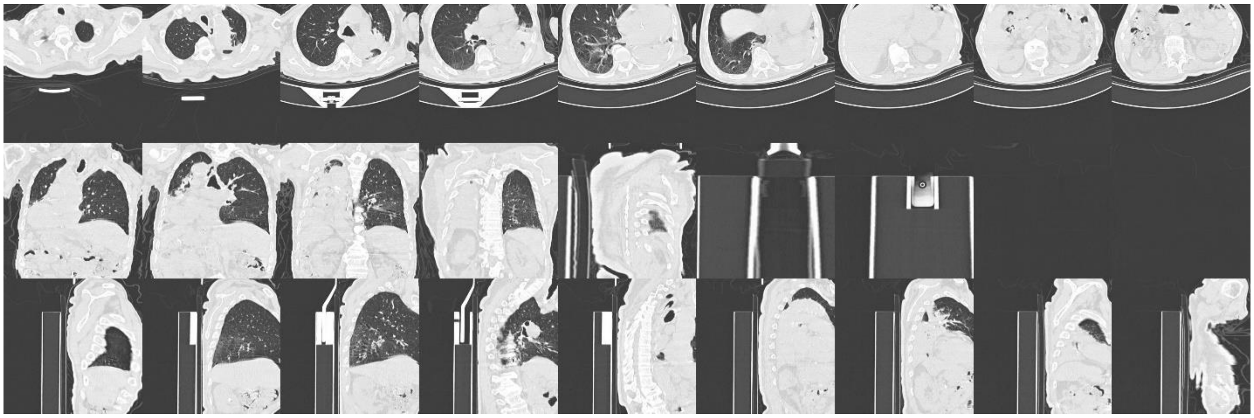

Breathing artefacts are common and seen in 113 scans (it is difficult for severely ill patients to maintain breath hold so this is to a certain extent inevitable). Here is patient 1092:

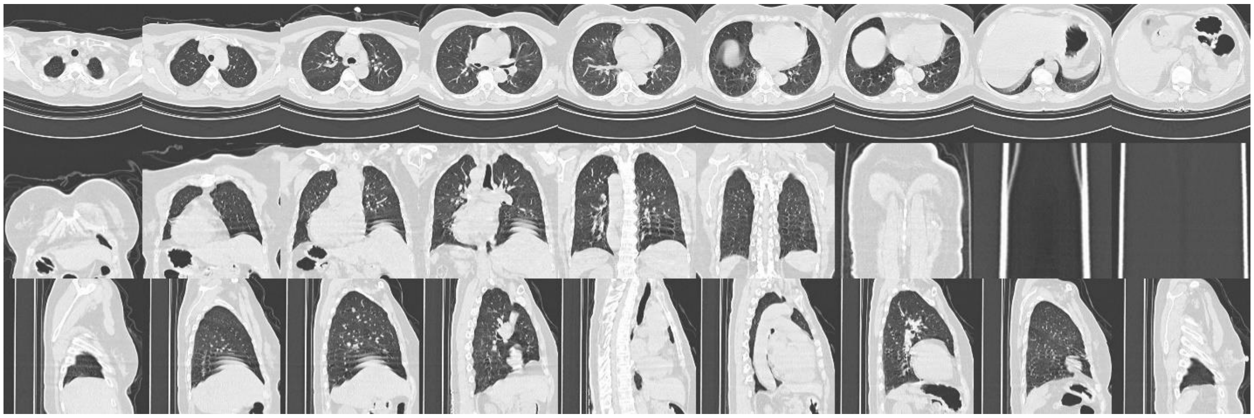

I can share my analysis of all scans with the authors.

The paper says that “All datasets were reconstructed with a slice thickness of 1.5-2 mm and an increment of 1.5-2 mm.” for HUST-UH and “a layer thickness of 1.25 mm, and a layer spacing of 1.25

mm" for HUST-LH. I think this is not correct. There are many scans with few jpgs and visually you see thick slices, eg patient 86:

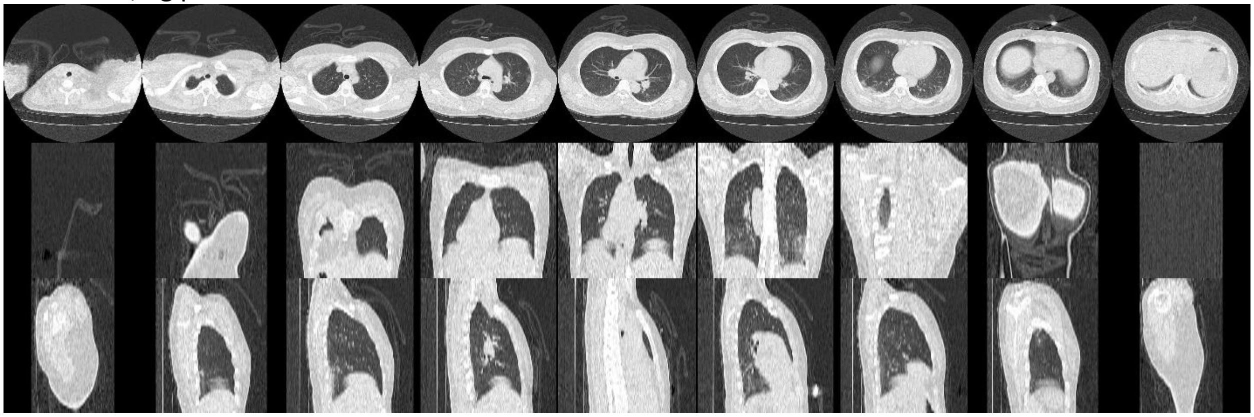

You see the vessels in the axial view, this looks like 5mm slice thickness to me. The low resolution in the axial also shows this. 86 scans have between 51 and 81 slices. Next the slices go from 121 to 530, I think a much wider range of slice thicknesses/spacing was used. This is important because for accurate image analysis one would resample to fixed usually isotropic resolution. Also slice based analysis as done in this paper is affected by slice thickness.

Some subjects have more than one scan. It was not clear to me at which time point all the provided clinical information was obtained in these cases. It would be good for all patients to provide when the clinical information and blood test outcome was obtained, same date of the CT or not?

In the paper, it was not really clear to me how the reference mild, severe, critically ill was made. It follows guidelines that use the CT, I believe, so classifying patients in groups determined by CT on the basis of CT is not so relevant. It may be more interesting to predict the course of disease or predict outcome of rt-PCR when this is not available (yet). This could be discussed in the paper.

The group labeled as controls are a mix of different subgroups. Some were scanned in a different time period (paper is not consistent in reporting this). More details should be provided. Which controls have community acquired pneumonia?

The suspects are not used in the experiments, but the website does provide PCR results for them. These may not be reliable (I found the CT for this group often inconsistent with the reported PCR). This should be explained better.

In the whole paper, CT is reported on as number of slices (2D) instead of studies. This should be reported in terms of studies. The number of images/slices is not relevant. You can always reconstruct the CT with thinner (and more noisy) slices. This does not provide more information.

Air branchogram: should be bronchogram

The paper is written with lots of enumerations in sentences. Summarizing results in tables would make the paper more readable.

The presented CT analysis is done per slice. Although such an approach is not uncommon in the literature, we know from comparative studies that 3D analysis gives better results. I would characterize

the presented approach as solid but not optimal. It is nice that the software is shared. Sharing software under a CC license is uncommon, better pick an open source software license, github provides many options. A major limitation of the code is that it is trained and tested on data from the same source. As two hospitals supplied data, it would make sense to train on data from one center and test on data from the other center. It would also be good to provide results for the different groups of scans, eg thick versus thinner slices.

Rebuttal 1

Detailed Responses to Reviewers' Comments

Comments raised by more than one reviewer

1. (From Reviewer #2) Moreover, the authors segment cases into negative, Type I, and Type 2, however do not reference which scale they are using or how their methods of review and type characterization (blinded radiologists?). Without a validated methodology to categorize severity the premise of this paper claiming clinical relevance to the clinician is minimized. The paper notes "researchers" and "clinicians" (could clinicians mean radiologists?) examined cases of HUST-19, however as the standard this should be done only by radiologists, who are the imaging experts. Moreover, what are the radiographic criteria that constitute "mild" and "severe"? This paper would be improved by correlating Type of pneumonia with morbidity and mortality outcomes.

(From Reviewer #3) In the paper, it was not really clear to me how the reference mild, severe, critically ill was made. It follows guidelines that use the CT, I believe, so classifying patients in groups determined by CT on the basis of CT is not so relevant. It may be more interesting to predict the course of disease or predict outcome of rt-PCR when this is not available (yet). This could be discussed in the paper.

(From Reviewer #4) For the study, the authors defined mild and regular forms as Type I, and severe and critically ill forms as Type II. Are there differences between them? Is the clinical treatment consistent? Whether they are combined analysis is reasonable?

We thank the reviewers for the question. We apologize for not being able to clearly describe how we classified COVID-19 cases in the original manuscript, which causes a confusion to the reviewers, thus leading to the above questions. In our study, suspected cases and different types of COVID-19 patients are actually clinically classified based the Guidance for Corona Virus Disease 2019 (6th edition) released by the National Health Commission of China (http://www.nhc.gov.cn/yzygj/s7653p/202002/8334a8326dd94d329df351d7da8aefc2/files/b218cfeb1bc54639af227f922bf6b817.pdf, or http://www.nhc.gov.cn/yzygj/s7653p/202002/8334a8326dd94d329df351d7da8aefc2.shtml), but not just based on CT features. This Guidance has been cited in a number of COVID-19 literature (e.g., *BMJ*, 2020,368, m1091, PMID: 32217556; *Lancet*, 2020, 395, 1054-1062, PMID: 32171076). In the Guidance, the elements including epidemiological history, clinical manifestation, SARS-CoV-2 nucleic acid test, symptoms and CT imaging are integratively considered for the clinical classification of suspected and confirmed cases. The information of clinical outcomes including morbidity outcomes (i.e. clinical classifications of COVID-19) and mortality outcomes was obtained from daily medical records of cases, and manually checked and confirmed by five experienced attending physicians. In our dataset, the numbers of mild and critically ill cases are quite limited. To avoid over-fitting in model training and prediction, we took mild/regular cases as Type I, and mild/critically ill cases as Type II. To avoid any confusion, the original Fig. 1c is removed. We revise the manuscript as below:

Page 16, started from paragraph 1 of the Methods, now changed,

"**COVID-19 case definitions and clinical classifications**

Patients were diagnosed as suspected cases of COVID-19 or confirmed cases with mild, regular, severe and critically ill forms according to the Guidance for Corona Virus Disease 2019 (6th edition) released by the National Health Commission of China[16].

Specifically, suspected cases were defined if they met the criteria: any of the following epidemiological history plus any two of following clinical manifestations, or all the three

clinical manifestations without clear epidemiological history. The epidemiological history included: (*i*) recent travel history in and around Wuhan, or other communities with reported cases within 14 days prior to the disease onset; (*ii*) contact history with COVID-19 infected case(s) (Positive nucleic acid test) within 14 days prior to the disease onset; (*iii*) contact history with patient(s) having fever or respiratory symptoms from Wuhan or surrounding areas, or reported communities within 14 days prior to the disease onset; (*iv*) a cluster of the disease onset. The clinical manifestations included: (*i*) fever and/ or respiratory symptoms; (*ii*) chest CT imaging evidence showing signs of COVID-19 pneumonia, including the appearance of multiple small patchy shadows, interstitial changes, and the peripheral lung abnormality at the early stage, the rapid progression to multiple focal or diffuse bilateral GGOs, and consolidations in severe cases; (*iii*) laboratory findings of normal or decreased number of leukocytes or lymphopenia at the early stage of disease onset.

If suspected cases had definitive SARS-CoV-2 nucleic acid positive evidence (RT-PCR positive for specimens, such as throat swabs), they were diagnosed as confirmed COVID-19. According to the Guidance[16], the morbidity outcomes of the confirmed COVID-19 cases were clinically classified as the following four forms based on illness severity: (1) Mild form, mild clinical symptoms without chest CT imaging signs of viral pneumonia; (2) Regular form, fever and respiratory symptoms with chest CT imaging signs of viral pneumonia; (3) Severe form should meet any of the following criteria, (*i*) anhelation (respiratory rate ≥ 30 breaths/ min), (*ii*) finger blood oxygen saturation ≤ 93% in ambient condition, (*iii*) arterial partial pressure of oxygen (PaO_2) / fraction of inspiration oxygen (FiO_2) ≤ 300 mmHg (1 mmHg = 0.133 kPa), with a formula of PaO_2/FiO_2 × [barometric pressure (mmHg)/ 760] for adjustment in high-altitude areas (> 1000 meters), and (*iv*) >50% lesions in chest CT imaging are clearly developed within 24 to 48 hours; (4) Critically ill form should meet any of the following criteria, (*i*) respiratory failure requiring mechanical ventilation, (*ii*) shock, and (*iii*) concurrently having other organ failure that needs intensive care unit (ICU) treatment.

In this study, morbidity outcomes are defined as mild/regular forms (Type I) and severe/ critically ill forms (Type II).

Data collection and preparation

The collection, use, and retrospective analysis of chest CT images, CFs and SARS-CoV-2 nucleic acid PCR results of patients were approved by the institutional ethical committees of HUST-UH (IRB ID: [2020] IEC (A001)) and HUST-LH (IRB ID: [2020] IEC (A001)). Informed patient consent was waived by the ethics committees due to the emergency of COVID-19. For all enrolled patients, their first sets of CT and CF data after admission were collected. The daily medical records of cases from HUST-UH and were manually checked and confirmed by three attending physicians (J.Z., a senior respiratory physician with > 10 years' experience; Q.Y, a senior physician with > 10 years' experience in infectious disease; J.W., a senior physician with > 10 years' experience in infectious disease), and the medical records of HUST-LH were checked and confirmed by two attending physicians (Y.Z., a senior respiratory and critical care physician with > 30 years' experience; H.P. a senior respiratory and critical care physician with > 20 years' experience). Clinical classifications (i.e. morbidity outcomes) of COVID-19 for each patient was determined and confirmed by aforementioned physicians according to the Guidance[16]. Any ambiguous or inconsistent records were resolved by discussion with these attending physicians.

In the Cohort 1, the data were from (*i*) patients receiving PCR nucleic acid testing and hospitalized between Jan 25 and Feb 20, 2020 in HUST-UH and HUST-LH; (*ii*) patients admitted to HUST-UH between Nov 14 and Nov 30, 2019, and diagnosed with community-acquired pneumonia; (*iii*) healthy cases from routine physical check-up. The 1170 patients included 775 patients from HUST-UH and 395 patients from HUST-LH (Fig. 1a). There were 222 control cases consisting of 112 patients with community-acquired pneumonia, 14 healthy cases, and 96 patients whose SARS-CoV-2 nucleic acid testing were negative and CT imaging showed no signs of COVID-19 infection. The 649 laboratory-confirmed COVID-19 patients were composed of 23 mild, 415 regular, 146 severe and 65 critically ill cases. The remaining 299 patients were the suspected cases.

Among these 1170 patients, 1000 patients had CT images that contained 272,927 CT slices. In the confirmed cases, there were 450 cured cases and 146 unknown cases (patients transferred to other hospitals during hospitalization). The remaining 53 deceased cases included 37 from critically ill forms, 12 from severe forms and 4 from regular forms. Due to the severe illness condition, only 17 deceased cases had CT examinations.

To further evaluate the accuracy of HUST-19, we prepared the Cohort 2 from (*i*) patients receiving RT-PCR nucleic acid testing and admitted between Feb 14 and Feb 29, 2020 in HUST-UH; (*ii*) patients admitted to HUST-UH between Aug 20 and Nov 30, 2019, and diagnosed with community-acquired pneumonia. The total 351 patients included 245 laboratory-confirmed COVID-19 patients and 106 control cases. For the morbidity outcomes, there were 1 mild, 181 regular, 56 severe and 7 critical ill cases in the confirmed cases. For the mortality outcomes, therewere 212 cured cases and 29 unknown cases. The remaining 4 deceased cases included 3 from critically ill form and 1 from severe form. The Cohort 2 was not used for model training."

Reviewer #2:

1. The authors should firstly be commended for their efforts in contributing an open-access repository of imaging and data during the pandemic – this will aid others in research. This paper, while interesting, can be categorized as of minimal clinical advancement in the field of radiology and medicine in its current form. It should be noted that imaging is not recommended by any leading radiological society to rule out COVID-19 pneumonia. Please refer to the American College of Radiology (https://www.acr.org/Advocacy-and-Economics/ACR-Position-Statements/Recommendations-for-Chest-Radiography-and-CT-for-Suspected-COVID19-Infection). Ultimately, clinicians are not treating the imaging, they are treating the patient. This is not to say that the

results are not impressive, but to note they are unlikely to contribute a fundamental clinical impact. Should there be additional impact of the algorithm, such as morbidity and mortality, this would be improved.

We are thankful for your comments and suggestions. We carefully revise the manuscript to add necessary descriptions on clinical classifications of suspected and confirmed COVID-19 cases. We add a new cohort as an independent dataset to evaluate the accuracy of HUST-19 for predicting the morbidity outcomes. We also implement a new model to predict the mortality outcomes. The fundamental clinical impact has been significantly improved. We address all your concerns shown as below.

2. The data on clinical feature patients (192 out of 1170) is limited.

We are sorry that we might not state this point clearly in the original manuscript. In our dataset, all patients have CF data, and we revise the corresponding description as "…All patients had CF data, and 1342 patients had both CT and CF data…" on Page 7, paragraph 1.

3. As above, because of the fundamental nature of COVID pneumonia, this paper is likely of minimal clinical relevance.

In the revision, the clinical relevance has been significantly improved. We add the descriptions (not clearly stated in the original manuscript, which caused a confusion explained above) on the clinical classifications of patients, which are based on the Guidance for Corona Virus Disease 2019 (6th edition) released by the National Health Commission of China. And the information of clinical outcomes including morbidity outcomes (i.e. clinical classifications of COVID-19) and mortality outcomes was taken from daily medical records after being manually checked and confirmed by five

experienced attending physicians are also added. The clinical morbidity outcomes and mortality outcomes are used to evaluate the prediction accuracy of HUST-19.

5. Lack of a proper gold standard by a radiologist (this paper notes “researchers” and “clinicians” labelled the CTs -unless these were radiologists this classification should be redone).

Before training individual CT slice-based models in HUST-19, 19,685 CT slices were manually labeled by four radiologists from the two hospitals. We add a new section in Methods to clarify this point as below:

Page 21, paragraph 1, added,

“**CT slice labeling and interpretation**

For training individual slice-based models in HUST-19, we manually labelled 19,685 CT slices exported from DICOM images after removing personal information for 61 COVID-19 patients and 43 control cases. During labeling, either clinical or laboratory findings were not accessed. Individual CT slices in JPEG format from cases of HUST-UH were labeled and interpreted by two radiologists (H.S., a senior thoracic radiologist with > 30 years' experience; Y.C, a radiologist with 5 years' experience in interpreting chest CT images). The CT slices from cases of HUST-LH were labeled and interpreted by two radiologists (HM.Z., a senior radiologist with 15 years' experience; HJ.Z, a radiologist with 5 years' experience in interpreting chest CT images). The radiologists independently labelled CT slices, and resolved any disagreements through discussion to achieve consensus and interpretation of CT imaging features. In total, we obtained 5705 NiCT, 4001 pCT and 9979 nCT slices.”

6. Limited dataset of clinical features.

We are sorry that we might not state the relevant point clearly in the original manuscript. In iCTCF, all enrolled cases have CF data.

7. Lack of reference or validated methodology to categorize Type I vs Type II.

We have added a new section “COVID-19 case definitions and clinical classifications” for the classification of different types of patients with COVID-19 pneumonia. In our dataset, the numbers of mild and critically ill patients are quite limited. We tried to construct the models for predicting these two types of cases, but found that over-fitting occurred and the models exhibited poor accuracy on testing datasets. Thus, we regard mild/regular and severe/critically ill forms as Type I and Type II cases, respectively.

8. Lack of radiographic features categorizing Type I vs Type II.

Suspected cases and different types of confirmed cases are clinically classified based on the Guidance mentioned above, not CT features alone. We have removed the original Fig. 1c and corresponding descriptions to avoid any confusion.

9. “radiographic dataset” should be referred to as “CT dataset” – most will interpret radiographic to mean chest xray, which is of prevalent use in the COVID pandemic.

We have deleted the word “radiographic” throughout the manuscript.

10. “Linear shadows” is not a radiographic term applicable to CT scans, please remove or rephrase.

We agree to your opinion, and delete this term from the manuscript.

11. Clarify who reviewed the CT scans and what their expertise is.

The information has been included in the new section “CT slice labeling and interpretation” on Page 21, paragraph 1.

12. Validate your Type reporting method or reference the validated methodology.

The methodology on clinical classification of patients has been referenced. Please refer to the newly added section in the Methods “COVID-19 case definitions and clinical classifications”.

13. Consider extending implications to morbidity and mortality outcomes, or length of stay, to make this more clinically relevant.

Our prediction of control, Type I and II cases in HUST-19 is actually the prediction of morbidity outcomes of COVID-19 (four forms according to the Guidance mentioned above). COVID-19 form for each patient was determined using daily medical records of patients and confirmed by five experienced attending physicians. We have now also obtained the information regarding mortality outcomes of patients if available (the mortality outcomes of patients who were transferred to other hospitals during hospitalization are not available, and regarded as “Unknown”).

14. Grammatical and spelling errors throughout.

We have tried our best to correct all grammatical and spelling errors throughout the manuscript.

15. Please correct the tense, varies between past and present.

In the revision, the inconsistency in the tense was cleared.

16. iCTCF needs to be defined on first use in the abstract.

We have spelled out the full name of iCTCF in the Abstract.

Reviewer #3:

1. The HUST-19 database is an excellent resource and the authors should be lauded for building such a resource under such difficult circumstances during a healthcare crisis in a region in lockdown. Sharing HUST-19 with a CC-BY-NC license makes a valuable contribution. It is important to provide the data to the research community as soon as possible. I do not know of any other dataset similar in size that is publicly available. Many groups are looking for data and developing software with small low-quality datasets.

We thank the reviewer very much for the comments. This is the reason that we develop this resource. We believe a high-quality dataset will be greatly helpful for developing software packages to predict morbidity outcomes (i.e. clinical classifications of COVID-19) and mortality outcomes of COVID-19 patients. We will continuously maintain and update this resource for academic research.

2. The main limitation of HUST-19 is the fact that the CT data is not provided in DICOM format. Providing jpg images of individual slices severely limits the possibilities for data processing. Reasons for this is that the pixel size is now not available, and the slice thickness is not provided (see also below). The 3D geometry of the scan can therefore only be guessed. I downloaded the 980 zip files and made a simple guess that each scan has a height of 500 pixels, relative to a size of 512x512 pixels per axial slice (note that many scans do not

have images that are 512x512, many are larger, and patients 110, 220, 230, 227, 208 contain a jpg of the scoutview of the study, these scoutview images should be removed). I resized all jpgs to 512x512 and constructed 3D volumes and made collages of axial, coronal and sagittal slices to analyze the scans.

In the revision, original CT images in both DICOM and JPEG formats are provided and available for downloading and sharing, under a CC-BY-NC 4.0 license. All patient-related information has been removed to ensure the anonymity.

3. In converting to jpg also the Hounsfield Units (a 16 bit scale) are lost (jpg is 8 bit). It is clear from visual inspection that the window-level at which the CT scan was displayed on the jpgs was not the same for every scan. Here are two examples, case 697 and 698:

In case 697 much of the mediastinum and abdomen is 255, pure white, while 698 uses a better window level probably close to a recommended setting of 1500/-500 W/L that is often used when inspecting the lung parenchyma on chest CT. With this setting soft tissues (muscle, fat) and bone can still be somewhat differentiated. As the window level setting is not known, it is not possible to accurately estimate Hounsfield Units from the data and many image processing algorithms rely on the fact that CT uses a calibrated density scale and will not work well on this data. Likewise, the deep learning algorithms trained on this data set will only work well on other CT data that is scaled, using this unknown procedure, to jpg images.

The CT examinations were performed by different radiologists. So the window level settings might be different. CT slices in JPEG format were directly exported from original

DICOM files. We directly used these JPEG slices, without any additional manipulation. We believe that providing DICOM files would help address this concern.

4. Inspecting the data, I found 135/980 cases with a visually clearly deviating window level (too bright mostly, there were also some cases with an abdominal window).

We have re-checked all CT images to resolve this problem. However, there are still some cases with a deviating window because all radiologists were very busy at that time due to an excessively large number of patients waiting for CT tests during the outbreak. Thus, the manipulation of CT systems might not be 100% perfect.

5. There are also 68 scans with missing slices. This includes 15 studies with only 2 jpgs, these should be removed and three studies with 10, 12, and 12 slices. An example of a case with missing slices is 73:

Note the discontinuity in the coronal and sagittal views.

We apologize for this inconvenience, which occurred during the upload of JPEG files into the online server. At that time, our network condition was quite limited. We have corrected this problem to ensure the consistency between JPEG and DICOM slices.

6. 6 studies are upside down. This is easily fixed of course.

We have corrected this problem.

7. I recommend to retrieve the DICOM data and share these. This should solve these issues. In its current form, the resource may also be useful, but much less so.

We agree to your opinion. Here, we confirmed that CT images in both DICOM and JPEG formats will be freely available for academic research. Our data size is 265.1 GB, and we are trying our best to upload all files to the online service, during the submission of this revision.

8. There are also limitations that would not be resolved even if DICOM would be available. 41 studies do not fully contain the lungs (field of view for the scan was not properly set. An example is patient 1160:

A severe example is patient 874:

We have discussed this problem with the radiologists from the two hospitals.

9. Breathing artefacts are common and seen in 113 scans (it is difficult for severely ill patients to maintain breath hold so this is to a certain extent inevitable). Here is patient 1092:

I can share my analysis of all scans with the authors.

Upon CT examinations, all patients are required to hold their breath. However, it is quite difficult for a number of patients to do so, due to their severe illness conditions. We directly use these images, without any additional manipulation.

Also, we are thankful for your analysis of all scans. We carefully provide a point-to-point response on this analysis. We do not manipulate or exclude any CT images. We directly use all of them.

10. The paper says that “All datasets were reconstructed with a slice thickness of 1.5-2 mm and an increment of 1.5-2 mm.” for HUST-UH

and “a layer thickness of 1.25 mm, and a layer spacing of 1.25 mm” for HUST-LH. I think this is not correct. There are many scans with few jpgs and visually you see thick slices, eg patient 86:

You see the vessels in the axial view, this looks like 5mm slice thickness to me. The low resolution in the axial also shows this. 86 scans have between 51 and 81 slices. Next the slices go from 121 to 530, I think a much wider range of slice thicknesses/spacing was used. This is important because for accurate image analysis one would resample to fixed usually isotropic resolution. Also slice based analysis as done in this paper is affected by slice thickness.

We discussed this problem with radiologists. The radiologists in HUST-UH frequently reconstructed CT images with 5-mm layer thickness and 5-mm layer spacing for a considerable proportion of patients to enable a faster examination during the difficult time of COVID-19 outbreak. We add corresponding descriptions in the manuscript shown as below:

Page 20, paragraph 2, changed,

“**Chest CT image acquisitions**

In HUST-UH, all patients underwent CT examinations in the supine position on one of the three CT systems: SOMATOM Definition AS+ (Siemens Healthineers, Germany), Discovery 750HD (GE Medical Systems, Milwaukee, WI) and TOSHIBA Activion 16 (Toshiba, Tokyo, Japans). The scanning range was set from the thoracic inlet to the diaphragm. The scan parameters were 128 × 0.6 mm or 64 × 0.6 collimation, 120 kV tube voltage, and 350 × 350 mm field of view. All datasets were reconstructed with a slice thickness of 1.5-2 mm and an increment of 1.5-2 mm. Due to the excessively large number of patients during the outbreak, CT images were frequently reconstructed with 5-mm layer thickness and 5-mm layer spacing for a considerable proportion of patients to enable a faster examination.

In HUST-LH, the chest CT scan of the patient was performed with a *uCT510* spiral CT scanner (United Imaging, China). The scanning range was set from the thoracic inlet to the diaphragm. The scan parameters are 32× 0.6 mm collimation, 120 kV tube voltage, and 350 × 350 mm field of view. All patients were in the supine position, and the patients were trained to breathe before the scan. During scanning, patients were asked to hold their breath. The scan ranged from the tip of the lungs to the lower edge of the costal angle. The original data were reconstructed into an image with 1.5-mm layer thickness and 1.2-mm layer spacing.

From the two hospitals, original CT images in DICOM format were obtained for all enrolled cases. To ensure patients' anonymity, a script was written in Python 3.7 to remove personal information and CT examination date from DICOM files."

11. Some subjects have more than one scan. It was not clear to me at which time point all the provided clinical information was obtained in these cases. It would be good for all patients to provide when the clinical information and blood test outcome was obtained, same date of the CT or not?

We confirm that only one CT scan is provided for each case if available. Due to limited medical resources, very few patients take multiple scans. The CT and CFs examinations were often conducted at different dates. In our dataset, a complete set of CFs examinations contain > 120 types of tests, which were often conducted in batches at multiple dates for patients. And some of CFs examinations were repeatedly conducted during hospitalization. Since patients' first set of CFs examinations after admission are the most integrative, we collected the first set of CT and CFs data after admission for all enrolled patients. For the time points, we have negotiated with the ethics committees, but our request was not permitted. Date information is not used for training, and excluding them has no influence on the reproducibility of the study.

12. The group labeled as controls are a mix of different subgroups. Some were scanned in a different time period (paper is not consistent in reporting this). More details should be provided. Which controls have community acquired pneumonia?

We thank the reviewer for the question. In the online server, we now add corresponding annotations as “Control (Healthy)” or “Control (Community-acquired pneumonia)” for corresponding cases.

13. The suspects are not used in the experiments, but the website does provide PCR results for them. These may not be reliable (I found the CT for this group often inconsistent with the reported PCR). This should be explained better.

Our dataset is continuously updated and refined. When we started to collect the data, these cases were clinically classified as suspected cases, without any SARS-CoV-2 nucleic acid evidence. During the revision, we have re-checked these cases’ medical records that were updated since our paper submission, and found that 51 of them were confirmed as positive cases after our paper submission. This information was also included in iCTCF for these suspected cases.

14. In the whole paper, CT is reported on as number of slices (2D) instead of studies. This should be reported in terms of studies. The number of images/slices is not relevant. You can always reconstruct the CT with thinner (and more noisy) slices. This does not provide more information.

We now change all CT images into CT slices throughout the manuscript. These slices in JPEG format are directly exported from DICOM files.

15. Air branchogram: should be bronchogram.

The original Fig. 1c has been removed, and corresponding descriptions including this term are also deleted.

16. The paper is written with lots of enumerations in sentences. Summarizing results in tables would make the paper more readable.

Due to time limitation, we have put many enumerations into the Methods, and simplified the main text in a more readable manner.

16. The presented CT analysis is done per slice. Although such an approach is not uncommon in the literature, we know from comparative studies that 3D analysis gives better results. I would characterize the presented approach as solid but not optimal. It is nice that the software is shared. Sharing software under a CC license is uncommon, better pick an open source software license, github provides many options. A major limitation of the code is that it is trained and tested on data from the same source. As two hospitals supplied data, it would make sense to train on data from one center and test on data from the other center. It would also be good to provide results for the different groups of scans, eg thick versus thinner slices.

We apologize that we cannot fully address this concern, because model training is quite time-consuming and we need at least two weeks to train additional models using the data from one center and testing on data from the other center, as well as uploading corresponding source codes to the online server. However, following the comments from other reviewers, we prepare a new cohort with 351 patients including 245

laboratory-confirmed COVID-19 patients and 106 control cases, as an independent dataset to test the accuracy of HUST-19.

Reviewer #4:

1. Almost all statistical test methods are t-test, whether the data distribution meets the statistical requirements?

We agree to your opinion that the normality of the CF data distribution should be tested prior to statistical analysis. In the revision, we use the Shapiro-Wilk test to evaluate the data distribution, and p-value < 0.05 denotes that the data distribution might not be normal. For normal distribution, the two-sided unpaired t-test is performed. Unexpectedly, we find that only 11 CFs follow the normal distribution. Thus, for CF data not following the normal distribution, a widely used non-parametric test, the Mann-Whitney U test, is performed. The original Supplementary Data 1 has been revised to add a column for p-values calculated from the Shapiro-Wilk test, and an additional column to show whether the data follows the normal distribution. The original Supplementary Data 3 is also updated to present the new results. We revise the manuscript as below:

Page 25, paragraph 3, added,

"**Statistical analysis**

The normality of the data distribution was evaluated by the Shapiro-Wilk test, using the stats.shapiro() function in Python 3.7. A threshold of p-value < 0.05 was set for data not following the normal distribution (Supplementary Data 1). For the 11 CFs following the normal distribution, the two-sided unpaired t-test was performed. For other CFs, the two-sided Mann-Whitney U test was performed using the stats.mannwhitneyu() function in Python 3.7. Mean value and standard deviation (S.D.) were calculated, and p-value $< 10^{-4}$ was considered as statistically significant."

2. I'm not sure if 10-fold cross-validations are enough to

demonstrate the credibility, is the suspected patients used as a validation dataset, whether an external validation dataset is needed?

We thank the reviewer for the comments. We agree to your opinion, and prepare a new cohort as an independent dataset to evaluate the accuracy of HUST-19 for predicting the clinical outcomes of COVID-19 patients. We revise the manuscript as below:

Page 18, paragraph 2, added,

"To further evaluate the accuracy of HUST-19, we prepared the Cohort 2 from (*i*) patients receiving RT-PCR nucleic acid testing and admitted between Feb 14 and Feb 29, 2020 in HUST-UH; (*ii*) patients admitted to HUST-UH between Aug 20 and Nov 30, 2019, and diagnosed with community-acquired pneumonia. The total 351 patients included 245 laboratory-confirmed COVID-19 patients and 106 control cases. For the morbidity outcomes, there were 1 mild, 181 regular, 56 severe and 7 critical ill cases in the confirmed cases. For the mortality outcomes, there were 212 cured cases and 29 unknown cases. The remaining 4 deceased cases included 3 from critically ill form and 1 from severe form. The Cohort 2 was not used for model training."

3. "Here, we used HUST-19 with the sensitive threshold to predicted 21,207 and 71 patients of 299 suspected cases to be COVID-19 negative cases, Type I cases, and Type II cases, respectively." should be "Here, we used HUST-19 with the sensitive threshold to predicted 21, 207 and 71 patients of 299 suspected cases to be COVID-19 negative cases, Type I cases, and Type II cases, respectively." Missing a comma.

We thank the reviewer for pointing this out for us. We have corrected this error.

4. Make sure references are up-to-date since this is a rapidly evolving topic. More information may be available at present.

We thank the reviewer for the comments. During the submission of our manuscript, we find that Dr. Zhang Kang et al. have published a paper in Cell. We summarize their major findings in Discussion. In Dr. Zhang's paper, the authors develop an AI system to predict patients with or without COVID-19 pneumonia, mainly using the CT imaging data. In our study, both CT and CF data has been used. Also, HUST-19 can predict both morbidity and mortality outcomes, whereas only morbidity outcomes can be predicted in Dr. Zhang's paper. In addition, only segmented CT slices of lung parenchyma in JPEG format are present in their study, and CT images in both DICOM and JPEG formats are provided. In both DICOM and JPEG files, all patient-related information and dates are removed to ensure the anonymity.

Rebuttal 2

Detailed Responses to Reviewers' Comments

Reviewer #2:

1. The authors have addressed most of the points raised with this revision. Unfortunately, the provided Severity rating scale link is no in English. My main comment was based around lack of description on who assessed the CT scans. Based on the updated revision, the authors have noted radiologists indeed reviewed the cases. I think given the guidelines differ vs those in the USA, the applicability may be somewhat more limited depending on the region of the reader.

We thank the reviewer for the comments. We carefully checked the COVID-19 Treatment Guidelines released by the National Institutes of Health (NIH) of USA (Updated on July 30, 2020, https://www.covid19treatmentguidelines.nih.gov/). In the USA guidelines, COVID-19 patients are categorized into five forms, including *Asymptomatic or Presymptomatic Infection* (SARS-CoV-2 positive without symptoms), *Mild Illness* (have COVID-19 signs/symptoms without shortness of breath, dyspnea, or abnormal chest imaging), *Moderate Illness* (have evidence of lower respiratory disease and $SpO_2 \geq 94\%$), Severe Illness (respiratory rate > 30 breaths/min, $SpO_2 < 94\%$, $(PaO_2/FiO_2) < 300$ mmHg), and/or lung infiltrates > 50%), and *Critical Illness* (have respiratory failure, septic shock, and/or multiple organ dysfunction).

In the Guidance for COVID-19 (6th edition) released by the National Health Commission of China, four major forms of COVID-19 patients are mild form (mild clinical symptoms without chest CT imaging signs of viral pneumonia), regular form (fever and respiratory symptoms with chest CT imaging signs of viral pneumonia), severe form (respiratory rate ≥ 30 breaths/min, $SpO_2 \leq 93\%$, $(PaO_2/FiO_2) \leq 300$ mmHg), and/or lung lesions > 50%) and critically ill form (respiratory failure, shock, and/or organ failure). The *Asymptomatic or Presymptomatic Infection* form was not included in the China Guidance. Thus, at least the definitions of *Mild Illness*, *Moderate Illness*, Severe Illness, and *Critical Illness* in the USA

guidelines are highly similar and consistent with mild, regular, severe and critically ill forms defined in the China Guidance, with nearly no significant difference. Thus, our iCTCF and HUST-19 can also be applied in other countries beyond China. We revised the manuscript as below:

Page 16, paragraph 1, added,

"…We carefully compared the China Guidance[16] and the USA Guidelines[40] for COVID-19, and found that the definitions of mild, regular, severe and critically ill forms of COVID-19 patients in the China Guidance[16] are highly consistent with *Mild Illness*, *Moderate Illness*, *Severe Illness*, and *Critical Illness* forms defined in the USA Guidelines[40]. Thus, iCTCF and HUST-19 can also be applied in other countries beyond China."

Page 19, paragraph 2, added,

"According to the COVID-19 Treatment Guidelines released by the National Institutes of Health (NIH) of the USA (Updated on July 30, 2020)[40], COVID-19 patients are categorized into five forms, including *Asymptomatic or Presymptomatic Infection*, *Mild Illness*, *Moderate Illness*, *Severe Illness*, and *Critical Illness*. Except for the suspected form in China and the *Asymptomatic or Presymptomatic Infection* form in the USA, the definitions of mild, regular, severe and critically ill forms in the China Guidance are highly similar to *Mild Illness*, *Moderate Illness*, *Severe Illness*, and *Critical Illness* forms in the USA Guidelines."

2. Would suggest the word "shadows" is removed from the revised methods as this is not in the CT radiology lexicon.

We agree to your opinion. We have changed the "shadows" into "GGOs", based on the advice from radiologists involved in this study.

Reviewer #3:

1. Please specify for the controls in Cohort 1 which cases are normal

and which are community acquired pneumonia.

We added a column entitled "Morbidity outcome" in Supplementary Data 2 to present the morbidity outcome for each patient for both Cohort 1 and 2. Patients with and without community acquired pneumonia were denoted as "Control (Community-acquired pneumonia)" and "Control", respectively.

2. Cohort 2 is named an independent dataset but this is not correct as the data in Cohort 2 is from the same hospitals as the data from Cohort 1. Just call it test set or validation set. List this as a limitation.

We changed the word "independent" into "validation" throughout the manuscript. We listed it as a limitation, and revised the manuscript as below:

Page 21, paragraph 1, changed,

"…The data in Cohort 2 was from the same hospitals as the data from Cohort 1. Thus, the Cohort 2 was not a fully independent dataset. The Cohort 2 was taken as a validation dataset and not used for model training."

3. The abstract says the combined analysis of CT and CF is superior to either one alone, but the results that show this are only in a supplement. I would suggest adding a table in the main paper giving the characteristics of the dataset, and another table with all the experiments and results.

Based on your comments, we have now added two tables in the main manuscript, including the Table 1 for the data characteristics of the Cohort 1 and Cohort 2, and the Table 2 for details on the performance evaluation of HUST-19

4. While I see the use of attempting to predict RT-PCR result from CT

and lab values, in situations where it is time-consuming to obtain these test results, and in situations where an initial test is negative but clinical suspicion of COVID-19 is high, I think there is no use for a system that predicts mild/regular versus either normal or severe/critical disease.

For the situations where an initial RT-PCR test is negative for a given patient, physicians/clinicians have to determine whether the clinical suspicion of COVID-19 is high or not, based on their experience, as well as other laboratory biochemical tests and/or CT results. However, such a judgement regarding COVID-19 would still be not confirmed without RT-PCR etiological results. Thus, in this situation, the classification of different COVID-19 types of patients is important as physicians/clinicians can accordingly implement clinical management and effectively allocate medical resources, especially in regions where the resources are limited. This can be well assisted by a predictive system, such as HUST-19 that can provide highly useful predictions using aforementioned lab and CT information (also used by physicians to make empirical diagnosis) to help physicians determine COVID-19 types of this given patient. If this patient was predicted to be severe/critically ill form, she/he should be isolated immediately and treated, and physicians/clinicians should order swab sampling for the patient consecutively to repeat the RT-PCR tests until a confirmed laboratory result is obtained. Because COVID-19 is a progressive disease, patients predicted to be mild/regular forms should also be isolated and clinically managed in a timely manner. Otherwise the severity of the disease might be worsened. Taken together, predictions of control/normal, mild/regular and severe/critically ill forms are all useful for physicians and medical systems towards an improved, timely clinical management (diagnosis and treatment) and an effective utilization of limited medical resources.

Reviewer #6:

1. Developed a nice piece of engineering work on Hybrid-learning for

UnbiaSed predicTion of COVID-19 patients (HUST-19) to predict morbidity and mortality outcomes. Unlike authors claim with the term "novel", I would suggest a nice piece of engineering works, since most of the methods were taken from the existing sources and integrated.

We are encouraged from your positive and helpful comments. We have changed the word “novel” into “engineering” throughout the manuscript.

2. How authors did come up with 13-layered CNN?

The 13-layer CNN was implemented based on the architecture of VGG-16, a classic CNN framework for image recognition. We have added corresponding descriptions in the manuscript as below:

Page 10, paragraph 3, changed,

“…To enable the slice-based prediction, we implemented a deep learning framework based on the architecture of VGG-16, a classic CNN framework for image recognition[24]. The original VGG-16 contained 16 weight layers including 13 convolutional and 3 fully connected (dense) layers, and too many parameters should be fine-tuned. To reduce model complexity and enable faster training, we only reserved 6 convolutional and 2 dense layers after extensive testing. The simplified CNNs contained 13 layers, including one input layer, 3 sets of dual convolutional and pooling layers (3 × 3), 2 dense layers, and one output layer (Fig. 3).”

3. Also, can we have clear explanation about how did those hyper parameters managed for this interesting task?

Sure, and we have added a new Supplementary Data 4 to present all pre-configured hyper parameters in both CNN and DNN models. We have revised the manuscript as below:

Page 28, paragraph 1, added,

"…CNN and DNN models were trained by minimizing cross entropy loss between final predictions and ground truth labels. During training, the Adam optimizer in Keras was adopted, and a decay factor *d* was used to control the learning rate at each epoch shown as below:

$$lr_i = lr \times \frac{1}{1 + d \times i} \quad (10)$$

Where lr was the initial learning rate and lr_i was learning rate at i-th epoch. Adjustable parameters such as the dropout ratio, initial learning rate, decay, and batch size were simultaneously optimized to improve the performance (Supplementary Data 4)."

4. How about custom CNN, inceptionNet (https://link.springer.com/article/10.1007/s13246-020-00888-x), or ChexNet?

To address your concerns, we have obtained the CNNs of Inception Net V3 and ChexNet directly from Keras, and used our CT data for model training to predict morbidity or mortality outcomes. We have now added a new Supplementary Fig. 3 to present ROC curves and confusion matrices for Inception Net V3 and ChexNet. It can be found that the two custom CNNs exhibited highly similar accuracies against HUST-19. We have added corresponding descriptions in the manuscript as below:

Page 16, paragraph 2, added,

"Besides HUST-19, we also implemented two additional open-source CNN frameworks, Inception Net V3[36] and ChexNet[37], for predicting morbidity or mortality outcomes using our CT data, respectively. The original architecture of Inception Net V3 contains 11 inception modules, which were truncated with only 3 inception modules and a grid size reduction module to accurately predict COVID-19 using chest X-ray images[38]. ChexNet was developed based on a 121-layer dense convolutional network (DenseNet-121)[39] to predict 14 types of thoracic diseases including pneumonia from chest X-ray images[37]. For predicting morbidity outcomes, we re-trained the

Inception Net V3 and ChexNet models using the Cohort 1, and their performance values on the Cohort 1 and 2 were present (Supplementary Fig. 3a-d). For predicting mortality outcomes, the same dataset used in HUST-19 was adopted for training the Inception Net V3 and ChexNet models. The 10-fold cross-validations were performed to evaluate the accuracy (Supplementary Fig. 3e, f). From the results, we found that the Inception Net V3 and ChexNet models achieved comparable accuracies against HUST-19 on both predicting morbidity and mortality outcomes (Supplementary Figs. 2a, c, e, 3). These results demonstrated that CNN-based models could accurately predict COVID-19 using CT data."

Page 28, paragraph 1, added,

"…In addition, the CNNs of Inception Net V3[36] and ChexNet[37] were directly obtained from Keras. Again, the Adam optimizer was used for model training, with an initial learning rate of 0.0001, a decay of 0.05, a batch size of 64 and epochs of 500. The Cohort 2 was adopted as a validation dataset."

5. Statistical analysis needs need to be extended. Take-home message must be clear from this section.

Clarity about the meaning of mean and standard deviation by taking sparsity (of the data) into account.

Based on your comments, the section entitled "Statistical analysis" was carefully improved. The meaning of mean and standard deviation was clarified by taking the data sparsity into account. We have revised the manuscript as below:

Page 28, paragraph 2, changed,

"For each of the 125 types of numerical CF, the normality of the data distribution was evaluated by the Shapiro-Wilk test, a commonly used normality test, using the stats.shapiro() function in Python 3.7. A threshold of p-value < 0.05 was set for a CF with data not following the normal distribution (Supplementary Data 1). For the 11 CFs with numerical data following the normal distribution, the two-sided unpaired t-test was performed using the stats.ttest_ind() function in Python 3.7

(Supplementary Data 3). For the remaining 114 types of numerical CFs with data not following the normal distribution, the two-sided Mann-Whitney U test, the nonparametric equivalent to the unpaired t-test, was performed using the stats.mannwhitneyu() function in Python 3.7 (Supplementary Data 3). Mean value and standard deviation (S.D.) were calculated, and p-value < 10^{-4} was considered as statistically significant. In statistics, mean and S.D. are measures of location and spread, respectively. When the data is sparse with extreme values, mean might not reflect the central location of data points, and S.D. will be high. Thus, mean and S.D. values calculated in this study could be only regarded as a reference. For multiple hypothesis testing correction, the adjusted p-value (< 10^{-3}) was calculated using the Benjamini-Hochberg method (Supplementary Data 3). For statistical comparisons of different types of patients with or without Udis, the two-sided chi-squared test was performed using the 2 × 2 table. The χ^2 was calculated and the p-value (< 0.05) was computed by the function of CHIDIST(χ^2, degree_freedom) in Excel. The degree_freedom was equal to 1 for each 2 × 2 table (Supplementary Data 3)."

6. How significant the test results are?

In this study, p-value < 10^{-4} was selected as the threshold to reserve statistically significant results. In the revision, we have further calculated the adjusted p-value using the Benjamini-Hochberg method, and found all revised results are significant with adjusted p-values < 10^{-3}. The Supplementary Data 3 has been updated to present adjusted p-values.

7. Can we take a look at statistical tests?

Sure. All statistical results were shown in Supplementary Data 3. Also, we have added a new Supplementary Fig. 1 to present the most significantly different results in each pairwise comparison.

8. Dataset collection is fairly large and happy to see that for computational scientists. Can they be available for research purpose?

Meaning, reproducible materials will be great.

Yes. All data sets have been archived at http://ictcf.biocuckoo.cn/. All data sets in iCTCF, and all computational models of HUST-19, Inception Net V3 and ChexNet are made available under a CC BY-NC 4.0 license.
